# Heterochromatin reorganization associated with the transcriptional reprogramming under viral infection in *Arabidopsis*

**DOI:** 10.1093/nar/gkag348

**Published:** 2026-04-17

**Authors:** Maria Luz Annacondia, Jinping Cheng, Vasti Thamara Juarez-Gonzalez, Juan Luis Reig-Valiente, Marco Catoni, German Martinez

**Affiliations:** Department of Plant Biology, Uppsala BioCenter, Swedish University of Agricultural Sciences, Uppsala 756 51, Sweden; Department of Plant Biology, Uppsala BioCenter, Swedish University of Agricultural Sciences, Uppsala 756 51, Sweden; Department of Plant Biology, Uppsala BioCenter, Swedish University of Agricultural Sciences, Uppsala 756 51, Sweden; Department of Plant Biology, Uppsala BioCenter, Swedish University of Agricultural Sciences, Uppsala 756 51, Sweden; Birmingham Institute of Forest Research, School of Biosciences, University of Birmingham, Birmingham B15 2TT, United Kingdom; Department of Plant Biology, Uppsala BioCenter, Swedish University of Agricultural Sciences, Uppsala 756 51, Sweden

## Abstract

Epigenetic mechanisms are key regulators of genomic integrity and genic expression. Emerging evidence shows that epigenetic dynamism is an important component of the transcriptional reprogramming during stress. Despite this, the overall stress-induced reprogramming of the different epigenetic marks and their targets is unknown. Here, we uncovered multiple epigenetic changes taking place during viral infection in *Arabidopsis thaliana* and their connection with gene expression. We find that cucumber mosaic virus (CMV) infection induces an overall reorganization of the repressive epigenetic marks H3K9me2, H3K27me3, and DNA methylation, which interact between them and are dynamic during infection. Overall, these epigenetic changes are involved in the reprogramming of the transcriptional program to adapt to the biotic stress and might ensure genome stability through the transcriptional control of transposable elements (TEs). Mechanistically, we demonstrate that the catalytic component of the Polycomb Repressive Complex 2 (PRC2) CURLY LEAF (CLF) mediates the transcriptional repression of genes gaining H3K27me3 during viral infection and that mutants on that component induce resistance against CMV. Additionally, we identify an interaction between the virus-produced protein 2b and CLF, pointing to direct interference of H3K27me3 homeostasis by CMV. Altogether, our results provide a complete picture of the epigenetic changes taking place at repressive marks that occur during viral infection stress and exemplify the overall dynamism of epigenetic regulation in eukaryotic organisms.

## Graphical Abstract

**Graphical Abstract:**
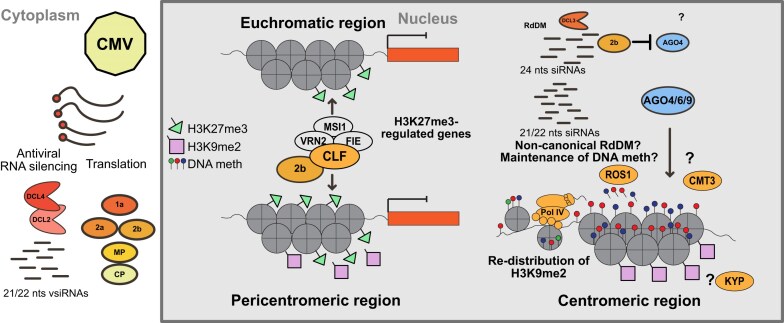

## Introduction

Eukaryotic organisms adapt to their environment by modulating their transcriptional program [1, 2].All An extreme case of environmental signals is stress, which usually induces a myriad of development defects due to the elicitation of the defense response [2]. In plants, biotic stresses, in general, and viruses, in particular, induce alterations that result from their hijacking of the host molecular machinery to complete their life cycle [3, 4]. To counteract viral infections, plants have several overlapping defense mechanisms that inhibit the accumulation of viral genomes and/or modulate gene expression [5–9]. Due to their dynamic nature, epigenetic mechanisms have been proposed as important regulators of the defense response during stress [10–12]. Nevertheless, how epigenetic mechanisms mediate this transcriptional control is poorly understood.

Epigenetic regulation comprises different mechanisms that are fundamental for the maintenance of genome stability [13–15] and the regulation of the transcriptional program in crucial biological processes [16–21], including the response against stress [10–12, 22]. These mechanisms include covalent and reversible marks that are introduced on genomic DNA and the histone proteins that make the nucleosome [23]. Genomic DNA can be covalently modified in the residue cytosine by a methyl group. In plants, this modification can be found in three sequence contexts that are maintained by independent machineries: CG, CHG (where H represents A, C, or T), and CHH [24]. DNA methylation is introduced in the genome by the activity of the RNA-directed DNA methylation pathway, which uses dsRNA transcripts generated by RNA polymerase IV (Pol IV) and copied by RNA-DEPENDENT RNA POLYMERASE 2 (RDR2) to produce DCL3-dependent 24-nt siRNAs that target Pol V transcripts (ref). This interaction between 24-nt siRNAs and Pol V transcripts recruits DOMAINS REARRANGED METHYLTRANSFERASE 2 (DRM2), which methylates cytosines in all three contexts [24, 25]. Of note, non-canonical versions of this pathway can also introduce DNA methylation [25–27]. DNA methylation is then maintained through DNA replication by the action of DNA methyltransferases, which are specific to each context: METHYLTRANSFERASE 1 (MET1), CHROMOMETHYLASE 3 (CMT3), and CMT2 maintain CG, CHG, and CHH methylation, respectively [15, 28, 29]. Genome-wide analyses of DNA methylation indicate their preference for centromeric and pericentromeric regions, where it is especially abundant at repetitive sequences [28, 30].

In addition to DNA methylation, plants also present posttranslational modifications of the histone tails forming the nucleosome [31, 32]. These modifications determine chromatin compaction defining euchromatic and heterochromatic regions that are characterized by low and high compaction, respectively [33]. Heterochromatin can be further divided into constitutive or facultative, which indicates whether chromatin is structurally silent or can respond to external effectors [33, 34]. These two statuses of chromatin compaction are mediated by histone methylation, in particular H3K9me2 and H3K27me3, which are associated with constitutive and facultative heterochromatin, respectively. H3K9me2 is highly enriched at TEs and repetitive sequences [35] and is mediated by members of the SUVH family of lysine methyltransferases, mainly SUVH4/KRYPTONITE (KYP). SUVH members can recognize methylated cytosines in CHG context, creating a feedback loop between DNA methylation and H3K9 methylation [32, 36, 37]. In contrast, H3K27me3 is not connected to DNA methylation and marks specific genes at their promoter and transcriptional start site [38]. H3K27me3 is catalyzed by the Polycomb protein complex Polycomb complex 2 (PRC2), which interacts with the PCR1-triggered ubiquitination of H2AK119 [37, 39, 40].

Among the different epigenetic mechanisms, the influence of DNA methylation over stress-induced transcriptional reprogramming has been widely studied in different plant-pathogen interactions such as the ones mediated by bacteria [41–44], fungi [45], nematodes [46], insects [47], viroids [48–50], and viruses [51–53]. Both hyper- and hypomethylation of gene regulatory [42, 54] and gene body regions [55] have been connected to transcriptional changes relevant to the response to stress. In addition, although less studied in plants, histone modifications have also been suggested as important players in the regulation of the defense response, with both histone de/acetylation and de/methylation playing a role in the transcriptional regulation of multiple stress-responsive genes [56–67]. Histone reorganization during stress is an important part of the stress-induced transcriptional reprogramming in multiple eukaryotic organisms, including mammals, *Neurospora*, and *Drosophila*, where repressive histone marks located in both facultative and constitutive heterochromatin (mainly the trimethylation of lysine 27 of histone H3, H3K27me3, and the di/trimethylation of lysine 9 of histone H3, H3K9me2/3, respectively) are redistributed under different types of stresses [68–73]. Nevertheless, the genome-wide dynamics of these two marks under stress or their connection to DNA methylation are underexplored in plants.

Since epigenetic regulation involves the interaction between multiple overlapping regulatory mechanisms, here, to fully understand the role and importance of these mechanisms, we studied the interaction between DNA methylation, the main repressive histone marks H3K9me2 and H3K27me3, and the transcriptional response to cucumber mosaic virus (CMV) infection in *Arabidopsis thaliana*. We discovered that H3K9me2 and H3K27me3 are more dynamic than DNA methylation and exert both a genome-protective role and a gene regulatory effect. In line with this role, we found that the catalytic component of the PRC2 complex CURLY LEAF (CLF) is resistant to CMV infection and a key player in the transcriptional regulation of CMV-responsive genes. Hence, the interaction between different epigenetic mechanisms is a key aspect of stress-responsive transcriptional reprogramming.

## Materials and methods

### Plant material, CMV infection, and phenotypic measurements

*Arabidopsis thaliana* (Columbia wild-type Col-0) was sown into potting soil (P-Jord, Hasselfors Garden, Örebro, Sweden). At the four-leaf stage, seedlings were selected by uniformity and carefully replanted into plastic pots (9 × 9 × 7 cm) with one plant per pot at temperature 20°C–22°C and 45% relative humidity. Plants were grown under a 16 h : 8 h, light : dark, photoperiod. The light was provided by FQ, 80 W, Hoconstant Lumix (Osram, Munich, Germany) with a light intensity of 220 μmol photons m^−2^ s^−1^. Mutant alleles used in this work include *drm1-2 drm2-2 cmt3-11* [74] (*ddc*), *ddm1-2* [75], *kyp-6* [76] (Salk_041 474), *nrpd1a-3* [77] (Salk_128 428), *clf-29* [78] (Salk_021 003), *ref6-1* [79] (Salk_001 018), *ago4-5* [80] (CS9927), *cmt3-11* [81] (CS16392), and *drm2-2* [82] (Salk_150 863). CMV infection was performed in plants at the four-rosette-leaf stage (1.04 stage from Boyes *et al.* 2001 [83]). Two leaves per *Arabidopsis thaliana* (Columbia wild-type Col-0) were rub-inoculated with a sap solution obtained by homogenizing 30-days infected *Nicotiana benthamiana* leaves in 0.1 M Na_2_HPO_4_ in the ratio of 1 g of plant tissues per 10 ml of buffer. Mock plants were rub-inoculated only with the 0.1 M Na_2_HPO_4_ buffer. *Nicotiana benthamiana* plants were infected with the three genomic RNAs of CMV strain FNY, previously obtained by *in vitro* transcription from plasmids containing the individual genomic sequences using the MAXIscript T7 Transcription Kit (Thermo Fisher). *Nicotiana benthamiana* symptomatic leaves were collected at 12 dpi and kept at −70°C as a viral reservoir. *Arabidopsis thaliana* samples were collected at 10 and 20 dpi. To determine the degree of the infection at a phenotypic level, measurements of the radius of the rosettes were taken at both 10 and 20 dpi. For that, two measurements for the rosette were taken, trying to take them in a cross shape as much as possible. The final measurements were calculated by doing the mean of the two numbers. Statistical significance was calculated using unpaired *t*-tests.

### DNA/RNA extraction and RT-qPCR

Genomic DNA for regular genotyping and bisulfite sequencing was extracted using the DNeasy Plant Mini Kit (Qiagen) following the manufacturer’s instructions. Total RNA was extracted using TRIzol reagent (Life Technologies) following the manufacturer’s instructions. For RNA sequencing, messenger RNA (mRNA) was purified with the NEB mRNA isolation kit (New England Biolabs). Extracted RNA was treated with DNase I (Thermo Fisher) and used to synthesize complementary DNA (cDNA) using the RevertAid First Strand cDNA Synthesis Kit (Thermo Fisher), following the manufacturer’s instructions. Then, cDNA levels were measured using the 5× FIREPol EvaGreen qPCR Mix Plus (ROX) (Solis Biodyne). Finally, relative accumulation was calculated using the “delta-delta method” formula (2^−<[ ΔCP sample - ΔCP control]^), where 2 represents a perfect PCR efficiency. *UBQ10* [primers used AAGCAGTTGGAGGATGGCAGAAC (forward) and CGGAGCCTGAGAACAAGATGAAGG (reverse)] was used as the housekeeping gene to which the levels of viral cDNA [primers used CTTCCAGAGATGCCTTCGAG (forward) and GGCAGTGCTTGTTCTTGACA (reverse)] were normalized. Statistical significance was calculated using unpaired *t*-tests. For each mutant, three biological replicates consisting of a pool of 8–10 plants and three technical replicates were used.

### Small RNA and RNA libraries preparation, sequencing, and analysis

Small RNA libraries were prepared using the NEBNext^®^ Small RNA Library Prep Set for Illumina^®^ (New England Biolabs), and each individual library was barcoded using the NEBNext^®^ Multiplex Oligos for Illumina^®^ kit (New England Biolabs). RNA libraries were prepared using the NEBNext^®^ Ultra™ II Directional RNA Library Prep Kit for Illumina^®^ (New England Biolabs) and each individual library was barcoded using the NEBNext^®^ Multiplex Oligos for Illumina^®^ kit (New England Biolabs). For both types of libraries, two (three for some RNA sequencing libraries) biological replicates consisting of pools of 12–20 plants per condition were sequenced. The obtained de-multiplexed libraries were adapter trimmed, and filtered by length (16–50 nt) and quality (Phred score: 20) using Trimgalore 0.6.1. For sRNA-seq analysis, reads were aligned to the *Arabidopsis* TAIR10 genome using bowtie [84] with the following parameters: -t -v2, allowing two mismatches. Library size was normalized by calculating reads per million of 18–28-nt genome-matching sRNAs.

RNA sequencing libraries were sequenced as paired-end 150 bp fragments in an Illumina NovaSeq 6000 at Novogene (Beijing, China). The obtained raw reads were trimmed using Trimgalore 0.6.1 for the removal of the adapter sequences. For expression analysis, paired reads were aligned to the *Arabidopsis* TAIR10 genome using STAR [85] with the following parameters: --outMultimapperOrder Random --outSAMmultNmax -1 --outFilterMultimapNmax 100 or default [10] -outSAMattributes NH HI AS NM MD --outSAMunmapped Within --outSAMtype BAM SortedByCoordinate --quantMode TranscriptomeSAM GeneCounts --outWigType bedGraph --limitBAMsortRAM 24000000000. The parameters -N10 or -N100 were used for the analysis of gene or TE expression, respectively. Afterwards, count reads per gene were obtained using HTSeq-COUNTS [86] with the following parameters: --mode union --stranded no --minequal 10 and --nonunique none. For TE expression analysis, paired reads were aligned to the *Arabidopsis* TAIR10 genome using STAR, allowing the mapping to at most 100 “best” matching loci with the following parameters: --outMultimapperOrder Random --outSAMmultNmax -1 --outFilterMultimapNmax 100, as previously used in Warman *et al.* 2020 [87]. The read counts per TE were obtained using HTSeq-COUNTS using the following parameters: --mode union --stranded no --minequal 0 and --nonunique all. The obtained count tables were used in DESeq2 [88] to infer significant expression with fit type set to parametric. All these tools were used on the Galaxy platform [89]. Volcano plots were created using the R package ggplot2 [90]. Principal component analysis was performed using DESeq2 built-in *plotPCA* function.

### Bisulfite library preparation and sequencing analysis

Bisulfite libraries of two bioreplicates per condition were produced from genomic DNA and sequenced as paired-end 150 bp fragments in an Illumina NovaSeq 6000 at Novogene (Beijing, China). The obtained raw reads were trimmed using Trimgalore 0.6.1 for the removal of the adapter sequences and 10 bases from 5′ ends. The remaining sequences were aligned to the *Arabidopsis* TAIR10 genome using Bismark [91], allowing one mismatch per 25 nt seed and keeping the forward and reverse reads independently mapped. Cytosine conversion rates were obtained using bismark_methylation_extractor; the first seven bases from the 50 end and 13 from the 30 end of each read were ignored. The mean conversion rate based on the cytosine methylation levels in the chloroplast genome for the four samples was 99.76%, and the estimated false-positive methylation rates were 0.24%.

DMRcaller [92] was used for DMR identification using the following parameters: 400 bp bins, 0.1 threshold for all sequence contexts, and the *computeDMRsReplicates* function for the management of bioreplicates. Public datasets analyzed for this paper are shown in Supplementary Table S4.

### Chromatin immunoprecipitation sequencing library preparation and sequence analysis

Five hundred milligrams of rosette leaves were chemically cross-linked using 1% formaldehyde. Nuclei were isolated from cross-linked material following a standard nuclei isolation protocol based on sucrose gradients as previously described [93]. Resuspended nuclei pellets were sonicated for 9 cycles of 20 s on and 45 s off at 4°C and high. Afterward, the immunoprecipitation (IP) was performed following a standard IP protocol and using the following antibodies: H3 (Reference: 07-690, Merck), H3K9me2 (Reference: pAb-060-050, Diagenode), and H3K27me3 (Reference: 07-449, Merck). The resulting immunocomplexes were purified with the GeneJET PCR Purification Kit (Thermo Fisher), following the manufacturer’s instructions. Finally, DNA libraries were prepared using the NEBNext^®^ Ultra™ II DNA Library Prep Kit for Illumina^®^ (New England Biolabs) and each individual library was barcoded using the NEBNext^®^ Multiplex Oligos for Illumina^®^ kit (New England Biolabs). ChIP libraries of two bioreplicates per condition were obtained from the immunoprecipitated DNA and sequenced as paired-end 150 bp fragments in an Illumina NovaSeq 6000 at Novogene (Beijing, China). The obtained raw reads were trimmed using Trimgalore 0.6.1 to remove the adapter sequences and 10 bases from 5′ ends. For genome-wide distribution analysis sequences were aligned to the *Arabidopsis* TAIR10 genome using bowtie2 with default parameters. BAM files were filtered for unique reads using the parameters -q20 -F255 -f0 × 2 and replicates were merged using samtools [94]. Genome coverage was calculated as the log_2_ fold change of the ratio between the coverage of H3K9me2 or H3K27me3 and the coverage of H3 using deeptools2 [95]. For genome-wide profile images, the RPKM-normalized value of H3 was subtracted from the RPKM value of either H3K9me2 or H3K27me3. Values for specific regions and quantitative analysis were retrieved using mapbed from bedtools [96]. Peak calling was performed using Sicer2 for each sample to its respective H3 control with the parameters: window size 200, fragment size 150, effective genome fraction 0.74, false discovery rate 0.01, and a gap size of 600 bp. Peak location and overlap were compared using the intersect tool from bedtools with a minimum overlap of 1 bp. Only peaks shared between the two replicates were considered true peaks for that specific treatment. Shared peaks were compared between samples using the intersect tool from bedtools to determine gain and loss peaks.

### Gene ontology term analysis

Gene ontology (GO) term analysis was performed using the *GO annotation search, functional categorization, and download* tool from the TAIR website (www.arabidopsis.org). Bar plots were created using the R package ggplot2 [90].

### Cytology

Immunostaining of *Arabidopsis thaliana* nuclei was performed as previously described [97]. In brief, 6-week-old (mock and CMV-infected) rosette leaves were collected and fixed in cold 4% paraformaldehyde in Tris–HCl buffer [10 mM Tris pH 7.5, 10 mM ethylenediaminetetraacetic acid (EDTA) and 100 mM NaCl] for 20 min, followed by two washes with ice-cold Tris–HCl buffer twice for 10 min each. Nuclei were isolated by chopping leaves in LB01 buffer (15 mM Tris–HCl pH 7.5, 20 mM NaCl, 2 mM EDTA, 80 mM KCl, 0.5 mM spermine, 0.1% Triton X-100) and filtered through a 30-μm CellTrics filter (Sysmex, Germany). The filtered nuclei were diluted 1:3 with sorting buffer (100 mM Tris pH 7.5, 50 mM KCl, 2 mM MgCl_2_, 0.05% Tween 20, 5% sucrose) and spotted onto microscopy slides to air-dry. The slides were post-fixed with 4% paraformaldehyde for 15 min at room temperature in PBS buffer (10 mM sodium phosphate, pH 7.0, 143 mM NaCl) and washed twice with PBS for 5 min each. Slides were blocked with 4% BSA for 30 min at 37°C in a moist box, followed by primary (anti-H3K9m2, C15410060, Diagenode, 1:500) and secondary (Alexa Fluor 488 Goat anti-Rabbit IgG1 Secondary Antibody, A21121, Invitrogen, 1:100) antibody incubation (all diluted in 1% BSA, 0.1% Tween 20, 1× PBS). After the antibody incubations, slides were mounted with 2 μg/ml DAPI and analyzed in a confocal microscope (Zeiss LSM780). Nuclei analyzed were selected according to their shape and integrity.

### Yeast two-hybrid assay

Yeast two-hybrid (Y2H) assays were performed following the manufacturer’s instructions for the Matchmaker GAL4 Two-Hybrid System 3 (Clontech, Mountain View, CA, USA). Bait and prey constructs were co-transformed into the yeast AH109 strain and plated on SD/–Leu/–Trp medium. After 2–3 days of incubation at 28°C, yeast colonies were collected, washed three times with 0.9% NaCl, and resuspended to a similar density. Serial 10-fold dilutions (1×, 10×, and 100×) were prepared and spotted onto SD/–Leu/–Trp and SD/–Leu/–Trp/–His/–Ade plates to test protein–protein interactions on selective medium.

### Protein co-immunoprecipitation

35S:GFP and 35S:2b-RFP, or 35S:GFP-CLF and 35S:2b-RFP were co-infiltrated into 4-week-old *N. benthamiana* leaves at a concentration of OD 600 = 0.6. Samples were collected after a 72-h incubation. 1.5 grams of tissue were ground in liquid nitrogen and homogenized with four volumes of protein IP buffer (50 mM Tris–HCl pH 7.5, 150 mM NaCl, 5 mM MgCl_2_, 0.1% NP-40, 0.5 mM DTT, 10% glycerol, 1 mM PMSF, 1 µg/µl pepstatin, and proteinase inhibitor cocktail). Samples were incubated on ice for 20 min and filtered through a 30-μm CellTrics filter (Sysmex, Germany). The flowthrough supernatant was centrifuged at 4000 rpm for 10 min at 4 °C. After centrifugation, 100 µl of the supernatant were kept as input, while the remaining sample was incubated with 25 µl of pre-washed (twice in IP buffer for 5 min) GFP-trap Magnetic Agarose (ChromoTek, gtma-20) for 4 h at 4°C. Beads were collected and washed five times with IP buffer containing 0.1% tween 20 for 5 min each. After this, the beads were resuspended in 1× PBS and boiled at 95°C for 10 min. Western blotting was performed with anti-GFP (Invitrogen A11122, 1:1000) and anti-2b antibody (Agrisera AS163981, 1:1000).

### Western blot

Approximately 500 μg of tissue was used to perform protein extraction using a standard protein extraction buffer (100 mM Tris, 150 mM NaCl, 0.5% NP-40, 1 mM EDTA, 3 mM DTT, proteinase inhibitor cocktail) in a 1:1 tissue-to-buffer ratio. Samples were vortexed, incubated with rotation for 30 min at 4°C, and centrifuged for 30 min. After this, the supernatant was separated, mixed with 2× loading buffer, boiled for 10 min, and loaded on a 15% acrylamide sodium dodecyl sulfate–polyacrylamide gel electrophoresis (SDS–PAGE) gel. The gel was run for 2 h at 100 V and transferred to a Roti-Fluoro PVDF membrane (ROTH). After transfer, the membrane was blocked for 1 h with PBS-T + 5% milk solution at room temperature and subsequently incubated with an anti-2b antibody (Agrisera AS163981, 1:1000) diluted in PBS-T + 1% milk solution overnight at 4°C. After this incubation, membranes were washed 3× for 5 min with TBS-T and incubated for 1 h with the secondary goat anti-rabbit IgG HRP-conjugated antibody (Agrisera AS09602, 1:10 000) diluted in PBS-T + 1% milk. After washing the membranes 3× for 5 min with TBS-T, the western blot was revealed using the Amersham ECL Prime Western Blotting Detection Reagent (ROTH) following the manufacturer’s instructions and using a LAS-3000 Imaging System (Fuji). Loading controls were obtained from incubating a section of the membrane corresponding to proteins with a molecular weight higher than 25 kDa with Ponceau S staining solution.

## Results

### Different epigenetic pathways control CMV susceptibility in *Arabidopsis thaliana*

**Figure 1.**
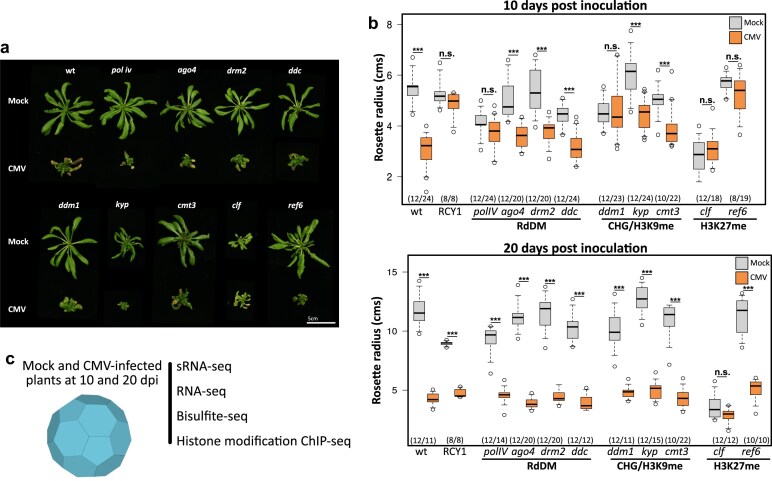
Epigenetic mutants show different susceptibility toward CMV infection. (**a**) Representative pictures of the mock and CMV-infected studied genotypes at 20 dpi. A scale bar representing 5 cm is shown as a reference. (**b**) Boxplot of the rosette diameter length in mock (gray boxes) and CMV-infected (orange boxes) plants at 10 dpi (top panel) and 20 dpi (bottom panel). Asterisks (***) indicate a *P*-value <.005; n.s. = “non-significant.” *P*-values were calculated using an unpaired t-test. Numbers between brackets indicate the number of individual plants (mock/infected) analyzed. (**c**) Experimental setup to explore the overall extent of CMV-induced epigenomic changes. Mock and CMV-infected tissues at 10 and 20 dpi were collected and used to generate whole-genome high-throughput sRNA-, RNA-, bisulfite-, and chromatin immunoprecipitation-sequencing. A cartoon depiction of CMV viral capsid is shown in blue.

Previous studies indicated that DNA methylation could be an important component of the plant viral-induced transcriptional response [98, 99]. To understand the contribution of epigenetic regulation to the tolerance against viral infection, we analyzed the susceptibility to CMV infection (the highly symptomatic subgroup I strain Fny-CMV [100], herein referred to as CMV) of mutants covering different epigenetic marks, including DNA methylation (*polIV, ago4, drm2*, and *ddc*), H3K9 methylation (*ddm1, kyp*, and *cmt3*), and H3K27 methylation (*clf* and *ref6*) homeostasis (Fig. 1a and b). As a positive control of CMV resistance, we included a transgenic line overexpressing the resistance gene RCY1 [101]. CMV-infected *Arabidopsis* plants are smaller and more compact than healthy plants [100]; hence, measuring the rosette radius is a good proxy of the viral symptomatology (Fig. 1a and Supplementary Fig. S1a). Quantification of the susceptibility at two different infection points, 10 days post-infection (dpi, onset of the viral symptomatology) and 20 dpi (advanced developed symptoms), showed marked tolerance to CMV infection in some of the mutant lines (Fig. 1b). At 10 dpi, the rosette radius of four different mutants covering the three epigenetic pathways under study (*clf, ref6, polIV*, and *ddm1*) did not show the expected significant differences between mock and infected plants, similar to RCY1 overexpressing lines (Fig. 1b top panel). At a later infection time (20 dpi), only *clf* retained a lack of significant difference between mock and infected plants, indicating a tolerance to CMV infection (Fig. 1b lower panel).

In agreement with an epigenetic component associated with CMV infection, infected plants showed a significant decondensation of H3K9me2 (Supplementary Fig. S1b). These changes were not attributed to a differential replication (Supplementary Fig. S2a and b) or accumulation (Supplementary Fig. S2c and d) of CMV in the mutant backgrounds. Overall, these results suggest that different epigenetic pathways, including DNA methylation, chromatin maintenance, and especially H3K27 methylation, play an essential (and previously uncharacterized) role in mediating tolerance against CMV infection.

### Transcriptional reprogramming during CMV infection is characterized by the activation of the defense response

To fully understand how DNA methylation and histone marks could play a role during CMV infection, we used high-throughput sRNA, RNA, whole-genome bisulfite (WGBS), and chromatin immunoprecipitation (ChIP, for the main heterochromatin determinants H3K9me2 and H3K27me3) sequencing from *Arabidopsis thaliana* plants infected with CMV at both 10 and 20 dpi (Fig. 1c).

**Figure 2.**
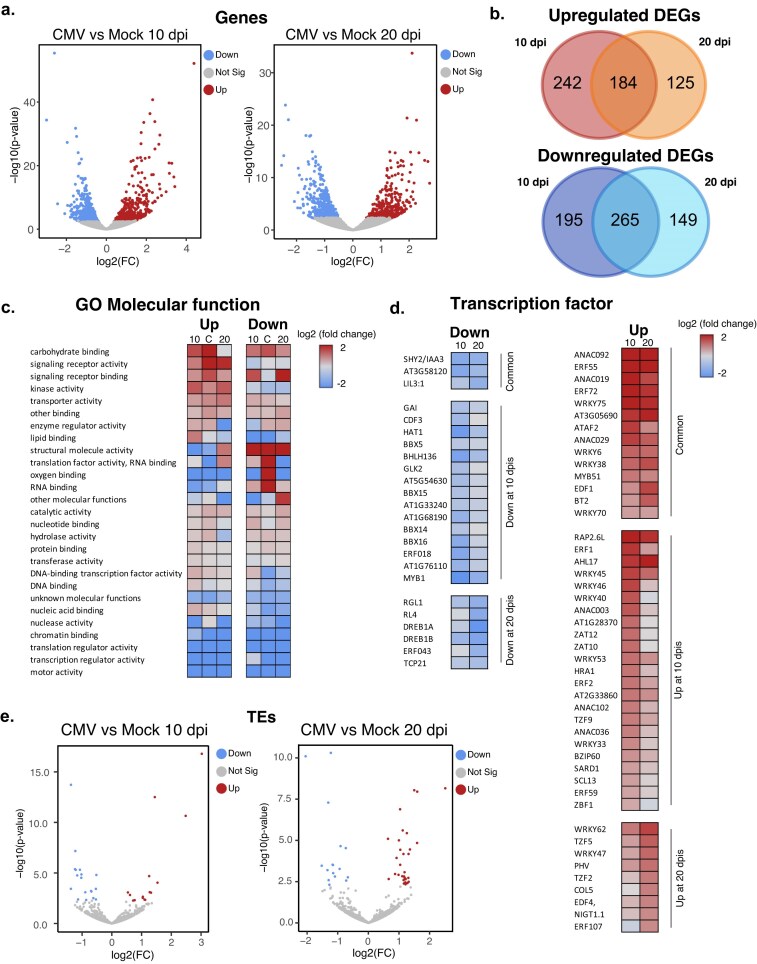
Transcriptomic reprogramming under CMV infection leads to the activation of the defense response. (**a**) Volcano plots showing the differentially expressed genes (DEGs) at 10 and 20 dpi. (**b**) Venn diagrams showing the overall overlap between 10 and 20 dpi upregulated and downregulated DEGs, respectively. (**c**) Heatmap for the GO categorization of genes according to their molecular function for DEGs showing upregulation (up) or downregulation (down) at 10, 20, or both times (labeled as 10, 20, or C, respectively). (**d**) Heatmaps of the transcription values [shown as log_2_ (fold change)] for TFs categorized as DEGs showing different patterns of transcriptional activity: downregulation (down) or upregulation (up) and at different time points as indicated. (**e**) Volcano plots showing the differentially expressed TEs at 10 and 20 dpi.

Previous studies of the transcriptomic response to CMV infection indicated strong deregulation of gene expression with multiple molecular functions affected that included metabolic processes, transcription factor (TF) binding, hormone signaling, and photosynthesis-associated processes [102, 103]. In agreement with previous works, our RNA sequencing analysis (Supplementary Fig. S3a) indicated that a substantial number of genes were differentially expressed (adjusted *P*-value <.05, herein DEGs) under CMV infection: 886 and 723 at 10 and 20 dpi, respectively (Fig. 2a and Supplementary Table S1). A similar number of genes were upregulated and downregulated at both 10 (426 and 460) and 20 (309 and 414) dpi, with a considerable overlap of misregulated genes in both the upregulated and downregulated fractions (56% average overlap between 10 and 20 dpi for upregulated and downregulated genes, Fig. 2b). GO categorization of CMV-responsive genes according to molecular function highlighted a preference of genes involved in metabolic regulation and signaling receptor activity being commonly enriched among upregulated genes, while genes involved in structural molecular activity, translation factor, oxygen, and RNA binding are commonly downregulated (Fig. 2c, enriched genes according to their overall genome representation). Our analysis highlighted a series of TFs that were identified as DEGs (upregulated or downregulated) and that had either general activity during infection or specific activity at either 10 or 20 dpi and might be important in the orchestration of the transcriptional response against CMV, such as HAT1 (downregulated at 10 dpi) [104] or WRKY70 (upregulated at both 10 and 20 dpi) [105] (Fig. 2d). We identified several TFs that were previously uncharacterized as DEGs under CMV infection and that are key for proper plant development, such as SHY2 (downregulated at 10 and 20 dpi) or several WRKY family members (6, 38, 51, and 75, all upregulated at 10 and 20 dpi) (Fig. 2d).

Of note, we could not identify any epigenetic pathway significantly misregulated at the time points studied, indicating that the transcriptional activity of the components of those pathways (if any) would take place before our sampling times (Supplementary Fig. S3b).

Different biotic and abiotic stresses have been associated with epigenetic changes, whose major targets are TEs [106]. Interestingly during CMV infection only a modest number of TEs experienced transcriptional changes under CMV infection (Fig. 2e). At 10 dpi only 15 TEs were transcriptionally upregulated and 18 TEs were downregulated, while at 20 dpi, 33 TEs were upregulated and 16 TEs were downregulated (Fig. 2e and Supplementary Table S2). Transcriptionally de-regulated TEs at both infection time points were classified as TEs with low CHH levels and/or regulated by the CHH-maintenance methyltransferase CMT2 (Supplementary Fig. S3c) [107]. Interestingly, the population of TEs transcriptionally deregulated at 10 dpi showed a less centromeric identity (characterized by shorter length and longer distance to the centromere) compared to the TEs deregulated at 20 dpi, indicating that the mechanisms regulating them might have different dynamics during the viral infection (Supplementary Fig. S3d). In summary, the results from our transcriptomic analysis reflected a genic transcriptional reprogramming characterized by the elicitation of markers of the defense response against stress and a lack of obvious epigenetic pathways affected at the times of infection analyzed.

### Overall DNA methylation gain during CMV infection

**Figure 3.**
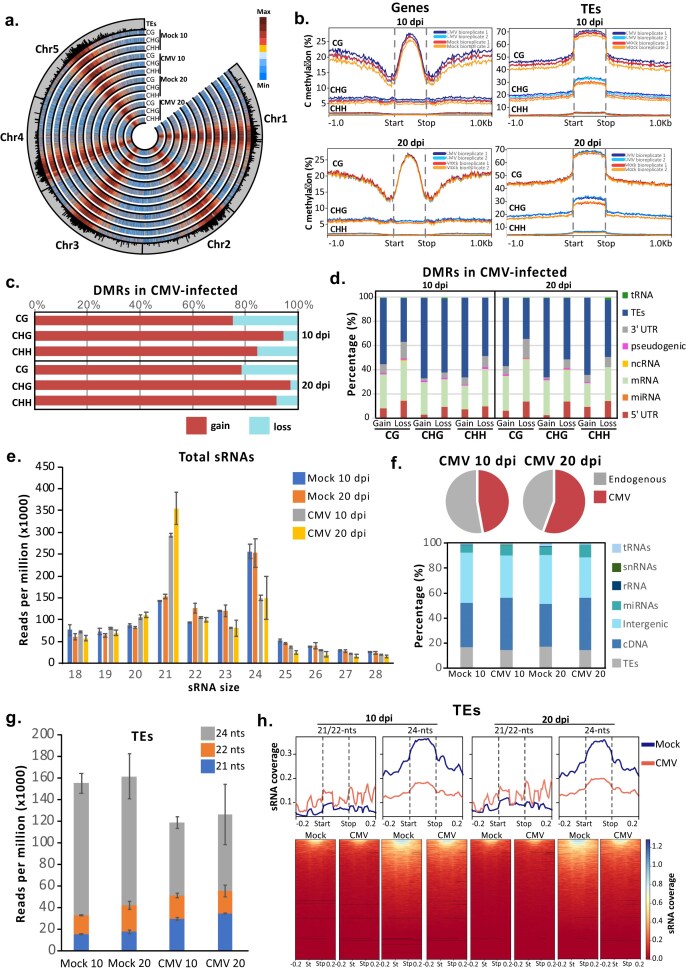
DNA methylation dynamics under CMV infection. (**a**) Circular plot showing the genome-wide levels of DNA methylation on each C methylation context on mock and infected samples at 10 and 20 dpi. The outside track shows the localization and genomic length of TEs in the *Arabidopsis* genome. (**b**) DNA methylation coverage profiles of each C methylation context for genes and TEs on mock and infected samples. (**c**) Overall presence of gain (hypermethylation) and loss (hypomethylation) differentially methylated regions (DMRs) in CMV-infected samples at 10 and 20 dpi. (**d**) Genomic categorization of gain (hypermethylation) and loss (hypomethylation) DMRs identified for each C methylation context on infected samples at 10 and 20 dpi. (**e**) Profile of the endogenous sRNA population from 18- to 28-nt in size, normalized to reads per million (×1000), in mock and CMV-infected samples at 10 and 20 dpi. Error bars show the standard deviation between two biological replicates. (**f**) Top: pie charts showing the overall presence of endogenous and CMV-derived siRNAs at 10 and 20 dpi; bottom: genomic categorization of the origin of endogenous siRNAs in mock and CMV-infected samples at 10 and 20 dpi. (**g**) Distribution of 21-, 22-, and 24-nt TE-derived siRNAs in mock and infected samples at 10 and 20 dpi. (**h**) Top: sRNA coverage profiles for 21/22- and 24-nt TE-derived siRNAs in mock (blue line) and CMV-infected (red line) samples; bottom: heatmap of the same sRNA coverage profiles represented in the top panel.

CMV has been previously linked to changes in DNA methylation as a consequence of the infection *per se* [98, 99] or through its viral silencing suppressor protein 2b [108, 109], which has been proposed to sequester RdDM-derived siRNAs. Our WGBS analysis (Fig. 3a) indicated that during CMV infection, at both 10 and 20 dpi, *Arabidopsis thaliana* experienced an increase of DNA methylation in all sequence contexts (Fig. 3b and Supplementary Fig. S4a and b), which is significant at both genes and TEs with the exception of genic CG context at 20 dpi (Supplementary Fig. S4a and b). This increase was more evident at TEs and was higher at 20 dpi, especially in the CHG context (Fig. 3b and Supplementary Fig. S4b). To further understand the connection between methylation changes and their genomic context, we identified differentially methylated regions (DMRs) [92]. This analysis allowed us to characterize a total of 2768 and 4438 DMRs at 10 and 20 dpi, respectively (Supplementary Fig. S4c). In accordance with the observed increased levels of DNA methylation, the majority of identified DMRs represent a gain of methylation and are enriched for the CHG context (Fig. 3c and Supplementary Fig. S4c). Overall, both gain and loss DMRs in the non-CG contexts are associated with TEs, while gain and loss DMRs in the CG context are mostly associated with genes, especially for the loss of DNA methylation (Fig. 3d). These changes most likely represented the enrichment of each of these sequence contexts in their preferential genomic context, with CG methylation being present at both genes and TEs, while CHG and CHH contexts spread only through TEs. DMRs between the two infection times were moderately conserved, with 18.2, 24.1, and 13.18% overlap for the CG, CHG, and CHH contexts, respectively (Supplementary Fig. S4d). Interestingly, analysis of the conservation of CMV-induced DMRs in different epigenetic mutants (including *met1, cmt3, cmt2, drm2, ddm1*, and *ros1*) [15, 110, 111] indicated that DMRs observed under CMV infection preferentially overlap with *ros1-* and *met1-*induced DMRs (Supplementary Fig. S4e and Supplementary Table S4).

DNA methylation is established by siRNAs derived from the RdDM pathway through its canonical and non-canonical form and maintained by the activity of context-specialized DNA methyltransferases together with the RdDM pathway [26, 112]. To understand the potential connection of siRNAs to the values of DNA methylation observed, we produced and analyzed high-throughput sRNA sequencing data. Our sRNA sequencing (Fig. 3e and Supplementary Fig. S5a) indicated striking differences between infected and non-infected tissues at both 10 and 20 dpi. These changes were characterized by a global decrease of 24-nt and an increase of 21-nt endogenous siRNAs (Fig. 3e). A majority of endogenous siRNAs were derived from genes and intergenic regions (Fig. 3f and Supplementary Fig. S5c and d), which reflected the changes observed in total siRNAs (Fig. 3e). Similar to our previous analysis of CMV virus-derived siRNAs (vsiRNAs) [113], CMV-infected *Arabidopsis thaliana* plants experienced an overaccumulation of vsiRNAs that increased with the infection time (Fig. 3f and Supplementary Fig. S5b).

Changes in sRNA accumulation were weakly connected to the loss of DNA methylation since hypomethylated DMRs showed a tendency to decrease 24-nt siRNAs (Supplementary Fig. S5e). Interestingly, these changes in 24-nt siRNA accumulation also took place globally over TEs (Fig. 3g and h), despite our observed increase in DNA methylation values over these loci (Fig. 3b). Importantly, compared to hypomethylated DMR regions, TEs gained 21-nt siRNAs, which could potentially be driving non-canonical RdDM (Fig. 3g and h). In brief, our data indicates that CMV infection led to changes in the global methylation profiles characterized by a gain of DNA methylation at all the different contexts and the presence of abundant hypermethylated DMRs at TEs, which were weakly connected to canonical RdDM activity.

### Dynamic reorganization of H3K9me2 and H3K27me3 during CMV infection

**Figure 4.**
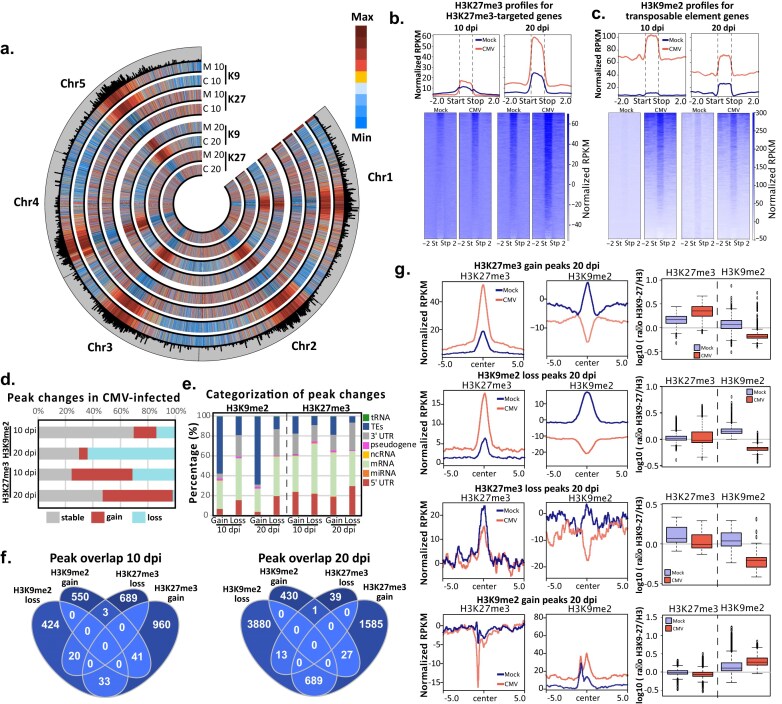
H3K9me2 and H3K27me3 are reorganized under CMV infection. (**a**) Circular plot showing the genome-wide levels of H3K9me2 (K9) and H3K27me3 (K27) on mock (M) and CMV-infected (C) plants at 10 and 20 dpi. Top: histone coverage profiles for H3K27me3-targeted genes (**b**) and transposable element genes (**c**) in mock (blue line) and CMV-infected (red line) samples; bottom: heatmap of the same histone coverage profiles represented in the top panel. Values represent RPKM normalized coverage for each mark with the subtracted values of H3 RPKM coverage. (**d**) Percentage of stable, gain, and loss H3K27me3 and H3K9me2 peaks in CMV-infected samples at 10 and 20 dpi. Peaks were defined in these categories according to their presence (defined as stable) or lack of presence (defined as gain if they were present only in CMV samples or defined as loss if they were present only in mock samples) in mock samples. (**e**) Genomic categorization of gain and loss peaks identified for each histone mark on infected samples at 10 and 20 dpi. (**f**) Venn diagram representing the analysis of the overlap of gain and loss H3K9me2 and H3K27me3 peaks at 10 (left) and 20 (right) dpi. (**g**) Left panel: H3K27me3 and H3K9me2 coverage profiles at H3K27me3/H3K9me2-gain and loss regions at 20 dpi in mock (blue line) and CMV-infected (red line) samples. Values represent RPKM-normalized coverage for each mark with the subtracted values of H3 RPKM coverage; right panel: box plots indicating the overall histone mark values (log_10_ of the ratio of histone mark/H3-input control). Box plots are Tukey, and *P*-values were calculated using an unpaired t-test.

Several components mediating histone homeostasis have been previously involved with the regulation of the stress response in plants. To understand the contribution of histone marks to CMV infection, we focused our analysis on the well-characterized repressive histone marks, H3K9me2 and H3K27me3, which cover the majority of heterochromatin (constitutive and facultative, respectively) in the *Arabidopsis thaliana* genome [114, 115]. Our ChIP-seq analysis largely confirmed this trend (Fig. 4a–c). CMV infection induces changes of both repressive marks at their targets (Fig. 4b and c), with an increase of H3K27me3 especially at 20 dpi (Fig. 4b and Supplementary Fig. S6a), and an increase of H3K9me2 at 10 dpi that decreases at 20 dpi (Fig. 4c and Supplementary Fig. S6b).

To understand in detail the regions affected by the changes at both histone marks during CMV infection, we identified peaks showing gain or loss of each histone mark under CMV infection (Fig. 4d) and analyzed their co-location with different genome features (Fig. 4e). On one hand, H3K9me2 showed a dynamic behavior, increasing with viral infection (29.5% of peaks were gain or loss peaks at 10 dpi versus 68.8% of peaks at 20 dpi). In line with the observed relative decrease of H3K9me2 at 20 dpi (Fig. 4c), at this time point 90.9% of the peaks showing dynamism were associated with the loss of this mark. Gain of H3K9me2 under CMV infection was restricted to very heterochromatic regions of the genome, with already high levels of this histone mark (Fig. 4g) and its associated high values of DNA methylation (Supplementary Fig. S6d). On the other hand, loss of H3K9me2 took place at regions with lower levels of H3K9me2 (Fig. 4g) and relatively low levels of DNA methylation (Supplementary Fig. S6d). Increased H3K9me2 was associated with a significant increase in DNA methylation in the CHG context at 10 and 20 dpi (Supplementary Fig. S6d). On the other hand, the loss of H3K9me2 was independent of DNA methylation (Supplementary Fig. S6d). In sum, H3K9me2 tended to be lost during CMV infection progression.

Conversely, H3K27me3 dynamism was observed throughout both infection time points, with 74.8 and 51.3% of peaks showing changes at 10 and 20 dpi, respectively (Fig. 4d). Gain and loss of H3K27me3 took place at similar levels at 10 dpi (59.5 and 40.5%), while at 20 dpi we observed a clear tendency to gain, with 97.8% of peaks showing this tendency (Fig. 4d), which agreed with the genome-wide profile of this mark at its target genes (Fig. 4b). As expected, H3K27me3 dynamics were completely independent of DNA methylation (Supplementary Fig. S6c). In summary, H3K27me3 was gained during CMV infection.

In line with their already described preferential association with genomic loci, under viral infection, gain of H3K9me2 was mainly located at TEs, while its loss took place at genes (Fig. 4e). On the other hand, both gain and loss of H3K27me3 were mainly associated with genes or their regulatory regions (Fig. 4e). In agreement with a loss of heterochromatic identity, reduced levels of H3K9me2 and H3K27me3 lead to higher levels of 21- and 22-nt siRNAs derived from these regions, potentially as a result of increased Pol II activity (Supplementary Fig. S6c and d). Interestingly, high levels of 21- and 22-nt siRNAs were also detected at regions gaining H3K27me3, possibly indicating the relative inability of this repressive mark to restrict Pol II transcription or its incorporation to reduce previously excessive Pol II transcription under heavy chromatin remodeling.

Furthermore, our data showed the existence of a compensation between H3K9me2 and H3K27me3 taking place under CMV infection, since at 20 dpi there was an overlap between regions that lost H3K9me2 and gained H3K27me3 (Fig. 4f and g). This interchange of H3K9me2 by H3K27me3 only took place at regions that had H3K9me2 identity since regions that lost H3K27me3 did not gain H3K9me2 and could retain their facultative heterochromatic identity (Fig. 4f). Although this compensation between heterochromatic marks was previously observed in mutants that massively lose chromatin or DNA methylation [116, 117], our data indicated that this compensation can also take place in a wild-type background. Altogether, our results indicate that histone marks are heavily reorganized during viral infection, with stronger changes and interactions between H3K9me2 and H3K27me3 at later infection times, which tend to be lost and gained, respectively, especially at later infection times.

### DNA methylation and repressive histone marks participate in the transcriptional reprogramming under CMV infection

**Figure 5.**
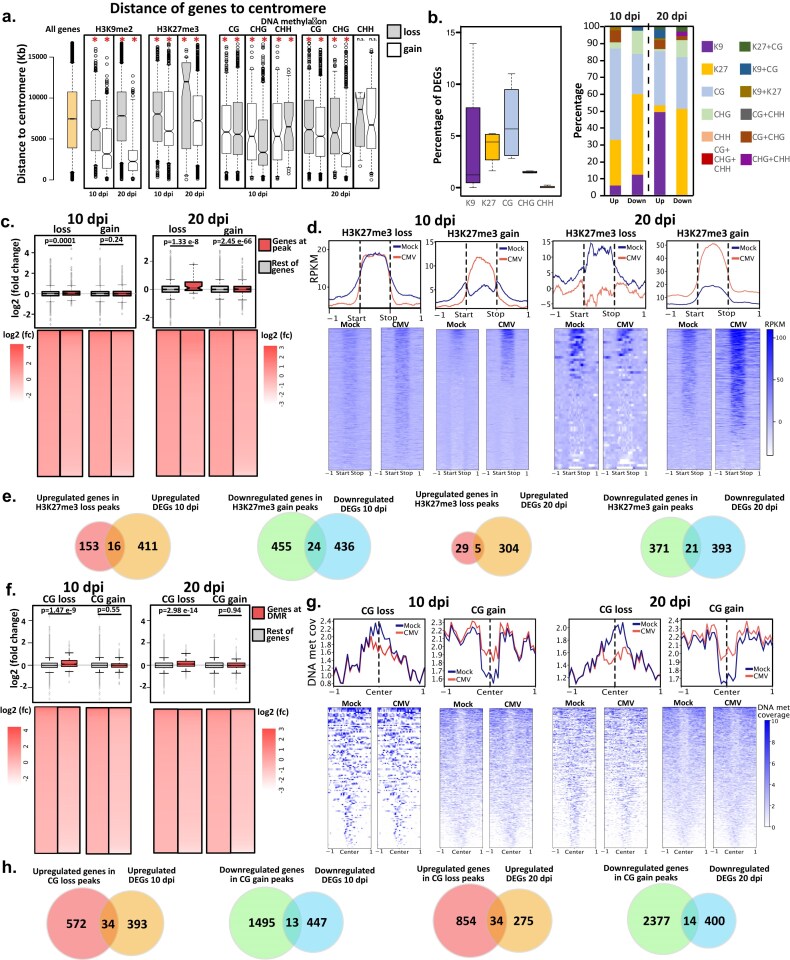
Epigenetic marks contribute to the transcriptional reprogramming under CMV infection. (**a**) Box plots depicting the overall distance to the centromere for all genes associated with each of the marks at the infection times indicated. The asterisk indicates *P*-value <.05 calculated through an unpaired t-test comparing the distance for each mark against the distance for all genes in the *Arabidopsis thaliana* genome (yellow box). (**b**) Left: Box plots indicating the percentage of DEGs within the overall genes associated with each epigenetic mark indicated in the color legend. Box plots were generated using 4 values for the percentage of DEGs within the overall number of genes associated with the indicated epigenetic mark under the different conditions analyzed: gain at 10 days, gain at 20 days, loss at 10 days, and loss at 20 days. Right: Association of the DEGs with each epigenetic mark under study here as indicated in the color legend. (**c**) Expression values [log_2_ (fold change)] for all genes associated with H3K27me3 loss and gain peaks at 10 and 20 dpi (colored in red) or the rest of the genes (all genes in the *A. thaliana* genome with the subtracted values for peak-associated genes). *P*-values are indicated on top of each comparison and were calculated through an unpaired t-test. (**d**) Top: histone coverage profiles for genes associated with a gain or loss of H3K27me3 peak at 10 and 20 dpi in mock (blue line) and CMV-infected (red line) samples; bottom: heatmap of the same histone coverage profiles represented in the top panel. Values represent RPKM normalized coverage for each mark with the subtracted values of H3 RPKM coverage. (**e**) Venn diagrams depicting the overlap between genes identified within an H3K27me3 peak and DEGs identified by RNA seq. The direction of the expression of the genes was considered to produce the overlaps (upregulation/downregulation of genes upon decrease/increase of H3K27me3, respectively). (**f**) Expression values [log_2_ (fold change)] for all genes associated with CG loss and gain peaks at 10 and 20 dpi (colored in red) or the rest of the genes (all genes in the *A. thaliana* genome with the subtracted values for DMR-associated genes). *P*-values are indicated on top of each comparison and were calculated through an unpaired t-test. (**g**) Top: DNA methylation coverage profiles for CG gain or loss DMRs at 10 and 20 dpi in mock (blue line) and CMV-infected (red line) overlapping with genes; bottom: heatmap of the same DNA methylation coverage profiles represented in the top panel. Values represent DNA methylation coverage. (**h**) Venn diagrams depicting the overlap between genes identified within a CG DMR and DEGs identified by RNA seq. The direction of the expression of the genes was considered to produce the overlaps (upregulation/downregulation of genes upon decrease/increase of CG methylation, respectively).

To understand the influence of DNA methylation, H3K9me2, and H3K27me3 over genic transcription under CMV infection, we analyzed the transcription level of genes that were directly associated with the gain or loss of these repressive epigenetic marks. We focus our analysis on genes that were directly associated with the gain or loss of H3K27me3 within their gene bodies, and genes that gained or lost DNA methylation and/or H3K9me2 within their gene bodies but also within a 1 kb window from their gene bodies, since both marks can control regulatory elements influencing gene expression [54]. Following that strategy, we detected 2192 and 11 356 genes associated with epigenetic marks at 10 and 20 dpi, respectively (Supplementary Fig. S7a). The genomic distribution of the genes associated with changes in the epigenetic marks followed the expected location of these marks over the genome, with changes in DNA methylation and H3K9me2 affecting genes significantly closer to the centromere and H3K27me3 affecting genes located further away from centromeres (Fig. 5a). Interestingly, following the exchange of repressive histone marks observed at later infection times, at 20 dpi H3K9me2 loss affects genes with slightly less pericentromeric identity and H3K27me3 gain follows the opposite trend (Fig. 5a). Several DEGs were found associated with each mark, with a preference for their association with H3K27me3, and different contributions of the other marks (Fig. 5b, Supplementary Fig. S7b, and Supplementary Table S3). Analysis of the correlation of gene expression with the presence of the different marks showed that indeed H3K27me3 (Fig. 5c) and CG methylation (Fig. 5f) showed the highest significant correlation with their gain or loss and the downregulation or upregulation, respectively, of the corresponding genes. Following our initial observations at the genome-wide level, changes in H3K27me3 (both loss and gain) at its target genes are more evident at 20 dpi compared to 10 dpi (Fig. 5d). H3K27me3 marked on average 3.9% of total DEGs (Fig. 5e). CG methylation variation over its target genes was not as strong as H3K27me3 (Fig. 5g), while it marked on average 6.3% of the overall number of DEGs (Fig. 5h). Regarding the rest of the marks, we only observed a correlation between the gain of H3K9me2 and the downregulation of gene expression at 10 dpi, and the gain of CHG methylation and the downregulation of gene expression at 20 dpi (Supplementary Fig. S7c–f). Intriguingly, CHH methylation did not correlate with gene expression changes at any infection time (Supplementary Fig. S7g and h).

**Figure 6.**
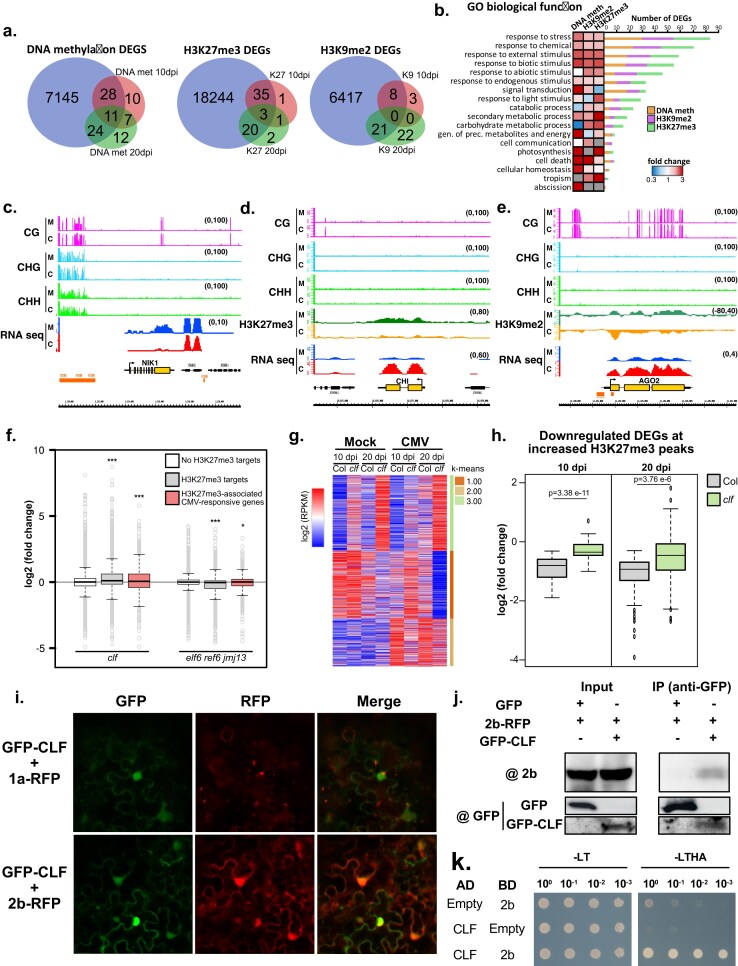
Epigenetic marks regulate multiple differentially expressed genes through different mechanisms and CLF is mechanistically involved in downregulating H3K27me3-associated genes during CMV infection. (**a**) Venn diagrams showing the overlap between DEGs associated with different epigenetic mechanisms and CMV-induced DEGs associated with the different epigenetic marks under study here (DNA methylation, H3K27me3, and H3K9me2). (**b**) Heatmap for the GO categorization of genes according to their biological function for DEGs associated with DNA methylation, H3K27me3, or H3K9me2. Number of genes associated with each category is shown on the right of the heatmap, with the colored number of genes associated with each specific mark. Genome browser screenshots showing the association of different viral-responsive DEGs previously identified as important in the viral infection process and associated with increased values of DNA methylation in the CG context at 10 dpi for NIK1 (**c**), decreased H3K27me3 at 20 dpi for CHI (**d**), and decreased H3K9me2 at 20 dpi for AGO2 (**e**). M = mock and C = CMV. (**f**) Box plots showing expression values [log_2_ (fold change)] for genes identified as non-H3K27me3 targets (white boxes), H3K27me3 targets (gray boxes), or H3K27me3 targets during CMV infection (red boxes) in *clf* and *elf6 ref6 and jmj13* mutant backgrounds. Asterisks show different *P*-values: *<.05, ***<.005. (**g**) Heatmap showing the RNA expression values (log_2_ of the RPKM values) for all H3K27me3-associated genes (as described in Zhang *et al.* 2007 [115]) under CMV infection in Col or *clf* genetic backgrounds in mock or CMV-infected conditions at 10 and 20 dpi as indicated. (**h**) Box plots showing expression values [log_2_ (fold change)] of DEGs downregulated by increased values of H3K27me3 under CMV infection in Col (gray boxes) and *clf* (green boxes) genetic backgrounds. *P*-values are indicated on top of each comparison and were calculated using an unpaired t-test. (**i**) Representative confocal images of the subcellular localization of *Nicotiana benthamiana* co-infiltrated 35s:GFP-CLF and 35s:1a-RFP or 35s:2b-RFP constructs. Merge of the GFP and RFP channels indicates the overlap of both signals. (**j**) Co-immunoprecipitation (Co-IP) assay showing the interaction of GFP-CLF and 2b-RFP transiently expressed in *Nicotiana benthamiana*. Protein extracts were immunoprecipitated with GFP-trap and resolved by SDS–PAGE. A 35s:GFP construct was used as a control for the specific interaction between CLF and 2b. (**k**) Y2H assay testing the interactions between 2b and CLF proteins. Yeast cells containing both the activating domain (AD) and binding domain (BD) vectors were grown on synthetic defined medium in the absence of Leu and Trp (SD/-LT, left panel) as a control and on synthetic defined medium in the absence of Leu, Trp, His, and adenine (SD/-LTHA, right panel) for the interaction test. Yeast cells were diluted 10-, 100-, and 1000-fold after culturing to a specific OD.

DEGs regulated by epigenetic marks were confirmed to be targets of their respective pathways since they were identified as differentially expressed (adjusted *P*-value <.05) in different combinations of mutants for genes involved in the homeostasis of DNA methylation (*mddcc* [117], *ago4* [118], and *polV* [119]; 68.5% of DNA methylation-associated DEGs), H3K27me3 (*clf* and *elf6 ref6 jmj13* [120]; 93% of H3K27me3-associated DEGs), and H3K9me2 (*kyp* and *ddm1* [121]; 54% of H3K9me2-associated DEGs) (Fig. 6a and Supplementary Table S4). The majority of overrepresented CMV-induced DEGs associated with epigenetic marks were classified as stress/stimulus-responsive genes according to their annotated GO biological function (Fig. 6b and Supplementary Fig. S8a). Interestingly, certain groups of genes were predominantly associated with some epigenetic marks, such as secondary metabolic processes (H3K27me3-associated) or cell death (DNA methylation- and H3K9me2-associated) (Fig. 6b). A similar result was obtained when genes were grouped by molecular function (Supplementary Fig. S8b). Reflecting our identification strategy, genes regulated by DNA methylation and H3K9me2 included genes with increased/decreased gene body and TE-like DNA methylation/H3K9me2 of elements surrounding their regulatory regions (Supplementary Fig. S8c and d). In comparison, H3K27me3 was only found associated with its presence in the gene body (Supplementary Fig. S8e). DEGs regulated by epigenetic marks included several important genes for the biology of viral infection, such as the previously identified virus-responsive genes NIK1 [122] (associated with DNA methylation, Fig. 6c); WAK1 [123], NHL10 [124], and CHI [125] (all associated with H3K27me3, Fig. 6d); and PUM5 [126], HSP70-2 [127], and the main antiviral AGO protein, AGO2 [128] (all associated with H3K9me2, Fig. 6e). In addition, CMV-responsive DEGs associated with epigenetic marks included other stress-responsive genes that have not been previously associated with viral infections, such as AT1G43910, AT1G13470, and AT1G67870, the lipid transfer protein EARLI1, the acyl-transferase protein CER26, and the FAD-binding Berberine family protein ATBBE10 (Supplementary Fig. S8c–e).

These results highlight the potential of epigenetic changes mediated by multiple epigenetic marks in regulating the transcriptional activity under CMV infection. In particular, we found that H3K27me3 and CG methylation changes were highly correlated with the expression of their associated genes.

### CLF represses gene expression during CMV infection and is a direct target of the viral silencing suppressor protein 2b

Next, we aimed to understand the mechanistic contribution of CLF (identified in our initial screening Fig. 1a and b) to the tolerance to CMV infection. H3K27me3 profiles under CMV infection showed a pattern compatible with an important role of this mark during CMV infection (Figs 4 and 5). Indeed, H3K27me3-associated genes during CMV infection were bona fide H3K27me3 targets since they behaved similarly to H3K27me3-targeted genes and were upregulated in a *clf* mutant background but downregulated in a triple *elf6 ref6 jmj13* mutant (Fig. 6f). To understand the role of CLF during CMV infection, we infected *clf* mutants with CMV and produced high-throughput RNA sequencing libraries at 10 and 20 dpi (Fig. 6g and Supplementary Fig. S9a–e). Our analysis of gene expression indicated that *clf* mutants showed increased expression level of H3K27me3 targets (Fig. 6g and Supplementary Table S5), which is significantly higher for DEGs that were downregulated in associated with increased H3K27me3 at both 10 and 20 dpi (Fig. 6h). Additionally, to understand the differences mediated by different regulators of H3K27me3, we performed RNA sequencing of mock and CMV-infected *ref6* mutants (Supplementary Fig. S10a). As expected from their opposite role in the regulation of H3K27me3 homeostasis, these two mutants showed different DEGs with minimal overlap, especially at 20 dpi (Supplementary Fig. S10b and c).

CMV produces five proteins that are involved in different aspects of the viral life cycle. In particular, the viral protein 2b has been shown to accumulate in the nucleus [129, 130], where it interacts with factors such as AGO4 [109]. Since CLF is a nuclear-accumulating factor, we wondered if any of the viral proteins might directly target this protein and, hence, affect H3K27me3 distribution. Co-infiltration of *Nicotiana benthamiana* with constructs driving the expression of CLF and the viral proteins 1a and 2b showed that, as previously described, only 2b has a nuclear accumulation that overlaps with CLF subcellular location (Fig. 6i). To test if 2b could be directly targeting CLF, we performed co-immunoprecipitation assays using tagged versions of 2b (2b-RFP) and CLF (GFP-CLF) in a heterologous system. Our analysis detected that 2b was indeed co-immunoprecipitated with CLF (Fig. 6j), suggesting that both proteins interact. Further confirming this interaction, Y2H tests demonstrated a strong interaction between 2b and CLF (Fig. 6k). Reanalysis of transcriptional data from plants infected with a 2b-deficient CMV strain (termed CMV-Δ2b) [131] showed that H3K27me3 targets were significantly upregulated in plants infected with this strain compared to a wild-type CMV (Supplementary Fig. S11). This result indicated that the effects observed on H3K27me3 levels might be a direct consequence of the nuclear accumulation of CMV-produced 2b protein and that the interaction between 2b and CLF might facilitate or increase H3K27me3 at some loci. In sum, our data shows that CLF is an important regulator of the transcriptional reprogramming taking place under CMV infection and that it negatively regulates the expression of genes targeted by H3K27me3 during infection. Furthermore, our analysis indicates that CLF is a direct target of the nuclear-accumulating viral protein 2b.

## Discussion

Epigenetic mechanisms regulate important aspects of cellular viability, such as the genome stability and/or its accessibility to the transcriptional machinery [132, 133]. A key aspect that defines these mechanisms is their dynamism, which provides a layer of versatility to rapidly adapt to environmental signals, including stresses [19–21, 134]. Indeed, the role of epigenetic marks in regulating gene expression under different biotic and abiotic stresses has been extensively described, with a particular emphasis on the role of DNA methylation in comparison to histone marks. Here, we have used CMV infection progression (a well-studied biotic stress) to analyze the genome-wide changes in multiple epigenetic marks and their connection to transcriptional activity (using both RNA and sRNA sequencing). We find that under CMV infection, the repressive histone marks H3K9me2 and H3K27me3 are more dynamic than DNA methylation and that H3K27me3 plays a role in tolerance to CMV infection. In line with this, we find that CLF (a catalytic component of the PRC2 complex) controls the downregulation of genes silenced by the increase of H3K27me3 during infection. Interestingly, the viral silencing suppressor 2b binds to CLF, pointing to the disruption of H3K27me3 as a direct consequence of viral protein accumulation. Our data also exemplifies the dynamism of epigenetic marks, which are extensively and functionally reorganized in a wild-type background subjected to biotic stress (such as the one used in our work), and not only in mutants for main components of epigenetic pathways.

Our analysis also adds more information about the particular role of the different epigenetic marks during stress. In our pathosystem, we did not identify major changes in DNA methylation, with only an overall increase in DNA methylation observed under CMV infection (Fig. 3). These changes, which occur mostly in the CHG context, might be attributable to the observed reorganization of H3K9me2 during CMV infection (Fig. 4) rather than to differential activity of the RdDM pathway. In line with this observation, most of the TEs differentially expressed under viral infection are not regulated by the RdDM pathway, and they either have low CHH methylation levels or their methylation is maintained independently of the RdDM pathway by CMT2. The lack of more widespread TE transcriptional reactivation during CMV infections is surprising due to the strong decrease of 24-nt TE-derived siRNAs observed (Fig. 3e). We find it plausible that TE transcriptional silencing under CMV infection could be a consequence of the strong H3K9me2 increase in centromeric and pericentromeric regions at both 10 and 20 dpi. Alternatively, DNA methylation increase could be attributable to either the action of a non-canonical version of the RdDM pathway using 21-/22-nt siRNAs to introduce DNA methylation or overall hyperactivity of the maintenance methylation pathways. Previous works have shown that CMV infection dramatically alters the sRNA landscape of the infected cells, leading to up to 50% of the cellular sRNAs deriving from the viral genomic RNAs [113]. These dramatic alterations of the overall siRNA profiles lead to the leaking of vsiRNAs into almost all endogenous AGO proteins, potentially affecting (directly and indirectly) multiple cellular processes [113]. Additionally, CMV viral silencing suppressor 2b is a known interactor of the PTGS machinery, sequestering sRNAs of different sizes and classes, including endogenous 24-nt siRNAs [113]. Interestingly, our analysis of the overlap between CMV-generated DMRs and DMRs associated with different epigenetic mutants showed an overlap with DMRs present in *ros1* and *met1* mutants (Supplementary Fig. S4d). This result might indicate that active DNA demethylation and restricted maintenance of DNA methylation, rather than restricted *de novo* DNA methylation, might be behind the observed changes in DNA methylation under CMV infection. We discard that Dicer-independent DNA methylation might occur in our tissues since no laddering of endogenous siRNAs (characteristic of this type of DNA methylation pathway) was observed in our sRNA sequencing data. Our data also highlights the need to consider the possibility that the dynamism in DNA methylation induced by multiple stresses could, at least partially, be the result of genome-wide histone reorganization.

Interestingly, we observed different needs for different epigenetic pathways at different stages of the infection. While at 10 dpi mutants in DNA methylation (*polIV*), H3K9 methylation (*ddm1*), and H3K27 methylation (*clf* and *ref6*) showed enhanced tolerance, at 20 dpi only *clf* retained this tolerance phenotype (Fig. 1b). In line with this result, H3K9me2 gain is more evident at 10 dpi (Fig. 4c), while H3K27me3 reorganization is strong at both 10 and 20 dpi (Fig. 4d). Several NB-LRR genes are located in centromeric and pericentromeric regions in the *Arabidopsis* genome [135] and are needed for the identification of the viral proteins and elicitation of the defense response [136]. It is plausible that the innate activation of NB-LRR proteins in mutants that affect constitutive heterochromatin (such as *polIV* and *ddm1*) confers tolerance to the virus infection at early time points. On the other hand, reorganization of facultative heterochromatin (marked mainly with H3K27me3) associated with the transcriptional reprogramming during stress might be needed at both 10 and 20 dpi. Interestingly, *clf* mutants resemble a lesion mimic mutant [137] for viral infection, which might highlight the role of H3K27me3 during viral response elicitation. Furthermore, our data identified a compensatory mechanism between H3K9me2 and H3K27me3 (Fig. 4e and f). This indicates that compensation of these heterochromatic marks is functional and takes place in a wild-type genetic background (under CMV infection) and is not only an artifact occurring in mutant backgrounds that have compromised chromatin, such as *mddcc* [117] or *ddm1* [116]. A similar functional compensatory mechanism between H3K9me2/3 and H3K27me3 has previously been observed in the silencing of chromosome X, which shows both high levels of H3K9me3 and H3K27me3 and complementation of both marks to mediate the X inactivation process [138].

Additionally, we found that the changes in all the repressive marks analyzed were connected to the transcriptional response during viral infection, and we found examples of genes regulated by both DNA methylation and repressive histone marks (Fig. 5). During the progression of CMV infection, both DNA methylation and H3K9me2 gradually associate with genes that have a stronger pericentromeric and centromeric location. At 20 dpi, H3K27me3 gain also leaks into pericentromeric locations, probably to compensate for the reorganization of H3K9me2 (Fig. 5a). Indeed, several DEGs are regulated by H3K27me3 (Fig. 6), the mark that better correlates with the direction of gene expression (Fig. 5c), which is expected since this epigenetic mark is key to regulating transcriptional networks [139]. DEGs regulated by H3K27me3 include the upregulated chitinase CHI (Fig. 6d), which is responsive to different viruses [125]; the upregulated gene ATBBE10, a FAD-binding Berberine protein member that is responsive to multiple biotic and abiotic stresses [140–142], epigenetically regulated [140], and whose overexpression is associated with resistance to bacteria [143]; and the downregulated gene CER26, a HXXXD-type acyl-transferase protein [144] that is positively regulated by histone acetylation and involved in stem cuticular wax biosynthesis [145]. Interestingly, we found that the reorganization of H3K9me2 is associated with the overexpression of AGO2, the main antiviral AGO protein, which is indeed a gene with pericentromeric location that contains multiple TEs in its 5′ regulatory region, a fingerprint of epigenetically regulated genes (Fig. 6e and Supplementary Fig. S8f). We found that downregulated DEGs under CMV infection (such as CER26) are targeted by CLF, indicating that this catalytic member of PRC2 is needed in the orchestration of the transcriptional reprogramming under viral infection and explaining the observed increased tolerance of CLF mutants to CMV. Rewiring of H3K27me3 and H3K9me2 could also be partially responsible for the transgenerational memory of stress that has been observed in plants exposed to different stresses [146–151] since both these marks can be partially inherited by the next generation [152, 153]. In addition, CMV infection improves plant resistance to different stresses, increasing plant longevity [154–156]. Since plant senescence has been connected to heterochromatin decondensation [157], it is plausible that the increase in H3K27me3 and H3K9me2 observed here could be a consequence of improved plant longevity caused by CMV infection.

We also observed a partial positive correlation between CG methylation changes and gene expression (Fig. 5). Several studies point to a very modest role of CG methylation in the control of gene expression in *Arabidopsis* [158, 159], which might suggest that our results could be an indirect effect of other epigenetic marks that accumulate in parallel to CG methylation [160]. A recent report linked variation in CG methylation (gene body methylation) to adaptation to different environments [159]. Our result might indicate a similar mechanism of plant adaptation to viral infection through CG methylation, despite our observed differences in gene expression correlation.

Interestingly, we identified an interaction between the CMV’s silencing-suppressor protein 2b and CLF. 2b is a well-characterized protein that is the pathogenicity determinant of CMV [161]. This connection to viral symptomatology has been linked to its silencing-suppressor activity [130, 162, 163], but also to its improved nuclear accumulation [129]. Our result might provide another clue on how 2b nuclear activities might influence CMV symptomatology. 2b interaction with CLF in the nucleus might affect H3K27me3 homeostasis, which we also demonstrated to be directly connected to the symptomatology of plants infected with CMV. Indeed, our reanalysis of transcriptomic data from plants infected with a 2b-deficient CMV strain showed increased expression of H3K27me3 targets, pointing to a 2b-mediated facilitation of CLF activity. Although this preliminary result needs further confirmation *in vivo*, future analysis of this interaction might shed light on the overall role of 2b in the nucleus and the whole array of processes that are affected by CMV infection.

Based on our results, we speculate that the hijacking of the cellular machinery by CMV leads to different simultaneous epigenomic consequences. First, a potential reorganization of peri- and centromeric constitutive heterochromatin induced by the initial NB-LRR-mediated recognition of viral accumulation might take place. This could also be caused by the previously characterized interference of CMV with the RdDM machinery caused by its viral suppressor of RNA silencing, the 2b protein, the generalized loading of vsiRNAs (and displacement of the endogenous siRNAs), or the activity of ROS1. These changes might lead to a preventive reorganization of H3K9me2 in pericentromeric and centromeric regions to avoid the transcriptional activation of TEs. This reorganization of constitutive chromatin might lead to two consequences for genic activity: (a) a transcriptional activation of pericentromeric genes and (b) a compensatory activity of H3K27me3 to silence excessive pericentromeric gene activity mediated (at least partially) by CLF. In parallel to this epigenetic activity at constitutive heterochromatin, the transcriptional reprogramming needed to fight against viral infection requires an extensive and continuous reorganization of facultative heterochromatin at both 10 and 20 dpi. Our work exemplifies that epigenetic changes associated with the stress response are not limited to DNA methylation and that the interplay between epigenetic marks during stress is extensive. Furthermore, our results provide a step further into understanding the interaction between plants and viruses and the dynamism of the epigenome during stress.

## Supplementary Material

gkag348_Supplemental_Files

## Data Availability

All raw and processed sequencing data generated in this study have been submitted to the NCBI Gene Expression Omnibus (GEO; https://www.ncbi.nlm.nih.gov/geo/) under accession number GSE247748.

## Notes

Present address: Department of Plant and Environmental Sciences, Copenhagen Plant Science Centre, University of Copenhagen, Frederiksberg 1871, Denmark
