# Supplementary material for: Heterochromatin reorganization associated with the transcriptional reprogramming under viral infection in Arabidopsis
Source: Nucleic Acids Res. 2026 Apr 17;54(7):gkag348. doi: 10.1093/nar/gkag348 (PMC13095653; doi:10.1093/nar/gkag348)
Supplement: gkag348_Supplemental_Files [file gkag348_supplemental_files.zip › Supplementary Materials Legends.docx]

**Supplementary Materials:**

**Supplementary Figure s1. a.** Representative pictures of CMV-induces symptomatology in *Arabidopsis thaliana.* Top left: rosette of mock treated plants at 20 dpi. Bottom left CMV-infected plants at 20 dpi. Right: Comparison of individual rosette leaves from mock or CMV-infected plants at 20 dpi. **b. Top:** Representative images of H3K9me2 immunostained nuclei from mock and CMV-infected leaves. **Bottom:** Box-plot showing the comparison of the area ratio of H3K9me2-stained chromocenters compared to the total nuclei area in mock (grey) and CMV-infected (white) samples. p-values were calculated using an unpaired t-test**.** Numbers within the boxes indicate the number of nuclei measured on each sample.

**Supplementary Figure s2. a-b.** RT-qPCR measured viral accumulation in CMV-infected plants at 10 (a) and 20 (b) dpi. For each mutant, three biological replicates consisting of a pool of 3-4 plant and three technical replicates were used. Error bars represent the standard deviation of the three biological replicates. p-values were calculated using an unpaired t-test**. c-d.** Western blot analysis of 2b accumulation in CMV-infected plants at 10 (c) and 20 (d) dpi for two different bioreplicates. Ponceau S-stained membranes for proteins bigger than 25 kDa is shown as a loading control.

**Supplementary Figure s3. a.** Principal component analysis of the RNA-seq samples. Two biological replicates were sequenced for each condition. **b.** Heatmaps showing the differential expression values (showed as log 2 (fold change)) between mock and CMV-infected samples for selected components of different epigenetic pathways. Asterisk in AGO2 denotes its identity as a DEG. **c.** TE classification according to the type of RNA-directed DNA methylation (RdDM) acting on the TE when TEs are silent (wt Col) as reported in Panda et. al., Genome Biology 2016 (107), for all TEs in the Arabidopsis genome or TEs differentially expressed at 10 and 20 dpi. **d.** Overall distance to the centromere (left) and length (right) for all TEs in the Arabidopsis genome (grey box) or TEs differentially expressed at 10 (green box) and 20 (orange box) dpi.

**Supplementary Figure s4. a.** Boxplot showing the percentage of each C methylation context in genes at 10 and 20 dpi. **b.** Boxplot showing the percentage of each C methylation context in TEs at 10 and 20 dpi. Boxplots are Tukey, and notches are +/-1.58 inter-quartile distance/sqrt(n). p-values were calculated using a t-test for unpaired values**.** **c.** Histogram of the number of hyper- and hypomethylated DMRs for each C context at 10 and 20 dpi. **d.** Venn diagrams indicating the overlap between DMRs at 10- and 20-dpi for each methylation context. **e.** Upset plots showing the overlap between CMV-associated DMRs and DMRs identified in different epigenetic mutants including *met1, drm2, cmt2, cmt3, ddm1* and *ros1*. Arrows indicate the highest number of DMRs overlapping between CMV and selected mutant (*ros1* and *met1* in the CG and CHG contexts, and *ros1* in the CHH context).

**Supplementary Figure s5. a.** Principal component analysis of the sRNA-seq samples. Two biological replicates were sequenced for each condition. **b.** Profile of CMV-derived sRNA populations from 18- to 28-nt in size, normalized to reads per million (x1000), in CMV-infected samples at 10 and 20 dpi. Error bars show the standard deviation between two biological replicates. **c-e.** Distribution of 21-, 22- and 24-nt siRNAs derived from intergenic (c), genic (d) and hypomethylated DMR (e) regions in mock and infected samples at 10 and 20 dpi.

**Supplementary Figure s6. a-b.** Histone coverage profiles of H3K27me3 and H3 ChIP for mock and CMV samples at 10 and 20 dpi for H3K27me3-targeted genes (**a**) and coverage profiles of H3K9me2 and H3 ChIP for mock and CMV samples at 10 and 20 dpi for transposable element genes (**b**). Values represent reads per genome coverage (RPGC) normalized to the *Arabidopsis thaliana* genome size (1x normalization). **c. Top:** box-plot depicting the values of DNA methylation for each sequence context at H3K27me3 gain (left) and loss (right) peaks. The lower panels show the distribution of 21-, 22- and 24-nt siRNAs derived from the same regions. **d. Top:** box-plot depicting the values of DNA methylation for each sequence context at H3K9me2 gain (left) and loss (right) peaks. Bottom: distribution of 21-, 22- and 24-nt siRNAs derived from the same regions is shown in the lower panels.

**Supplementary Figure s7. a.** Histogram showing the number of genes associated with loss (L) or gain (G) for each epigenetic mark under study at 10 and 20 dpi. The red section for each bar indicates the number of DEGs in each category. **b.** Association of all DEGs with epigenetic marks. The graph is similar to Fig 5b but includes all DEGs not associated with epigenetic marks (color in grey). **c.** Expression values (log 2 (fold change)) for all genes associated with H3K9me2 loss and gain peaks at 10 and 20 dpi (colored in red) or the rest of the genes (all genes in the *A.thaliana* genome with the subtracted values for peak-associated genes). Asterisks indicate the level of significance: *<0.05, ***<0.005. **d.** Top: histone coverage profiles for genes associated with a gain or loss H3K9me2 peak at 10 and 20 dpi in mock (blue line) and CMV-infected (red line) samples; bottom: heatmap of the same histone coverage profiles represented in the top panel. Values represent RPKM normalized coverage for each mark with the subtracted values of H3 RPKM coverage. **e.** Expression values (log 2 (fold change)) for all genes associated with CHG loss and gain peaks at 10 and 20 dpi (colored in red) or the rest of the genes (all genes in the *A.thaliana* genome with the subtracted values for DMR-associated genes). Asterisks indicate a p-value <0.05 (*) or <0.005 (***). p-values were calculated using an unpaired t-test. **f.** Top: DNA methylation coverage profiles for CHG gain or loss DMR at 10 and 20 dpi in mock (blue line) and CMV-infected (red line) samples overlapping with genes; bottom: heatmap of the same DNA methylation coverage profiles represented in the top panel. Values represent DNA methylation coverage. **g.** Expression values (log 2 (fold change)) for all genes associated with CHH loss and gain peaks at 10 and 20 dpi (colored in red) or the rest of the genes (all genes in the *A.thaliana* genome with the subtracted values for DMR-associated genes). p-values are indicated on top of each comparison and were calculated through an unpaired t-test. **h.** Top: DNA methylation coverage profiles for CHH gain or loss DMR at 10 and 20 dpi in mock (blue line) and CMV-infected (red line) samples overlapping with genes; bottom: heatmap of the same DNA methylation coverage profiles represented in the top panel. Values represent DNA methylation coverage.

**Supplementary Figure s8. a-b.** Heatmap for the gene ontology (GO) categorization of genes according to their biological (a) and molecular function (b) for DEGs associated with DNA methylation, H3K27me3 or H3K9me2. **c-e.** Genome browser screenshots showing the association of different DEGs with increased or decreased values of DNA methylation (c), DNA methylation and H3K9me2 (d) and DNA methylation and H3K27me3 (e). M=mock and C=CMV. **f.** Physical localization of AGO2 in the Arabidopsis thaliana chromosome 1 (Chr1). The genomic environment of AGO2 is shown in the highlighted box, with emphasis in the two TEs spanning its 5’ regulatory region.

**Supplementary Figure s9. a.** Principal component analysis of the Col-0 and *clf* genotypes*,* mock and CMV-infected at 10 and 20 dpi RNA-seq libraries. Three biological replicates were sequenced for each condition. **b.** Volcano plots showing the differentially expressed genes (DEG) at 10 dpi in Col-0 and *clf* genetic backgrounds. **c.** Venn diagram showing the overall overlap between Col-0 and *clf* DEGs at 10 dpi. **d.** Volcano plots showing the differentially expressed genes (DEG) at 20 dpi in Col-0 and *clf* genetic backgrounds. **e.** Venn diagram showing the overall overlap between Col-0 and *clf* DEGs at 20 dpi.

**Supplementary Figure s10. a.** Principal component analysis of the Col-0 and *ref6* genotypes*,* mock and CMV-infected at 10 and 20 dpi RNA-seq libraries. Two/three biological replicates were sequenced for each condition. **b.** Volcano plots showing the differentially expressed genes (DEG) at 10- and 20 dpi in Col-0 and *ref6* genetic backgrounds. **c.** Venn diagram showing the overall overlap between Col-0 and *ref6* DEGs at 10 dpi. **d.** Venn diagram showing the overall overlap between Col-0 and *ref6* DEGs at 20 dpi.

**Supplementary Figure s11.** Boxplot showing the expression level (log 2 fold-change) for H3K27me3-targeted genes in plants infected with CMV or the 2b-deficient strain CMV- Δ2b. p-value was calculated using an unpaired t-test.

**Supplementary Table 1.** Significant differentially expressed genes under CMV infection at 10 and 20 dpi.

**Supplementary Table 2.** Significant differentially expressed TEs under CMV infection at 10 and 20 dpi.

**Supplementary Table 3.** DEGs positively associated with epigenetic mark dynamism.

**Supplementary Table 4.** Public datasets used in this analysis.

**Supplementary Table 5.** Significant differentially expressed genes under CMV infection at 10 and 20 dpi in the Col-0 and *clf* genetic backgrounds.
