# Supplementary figures and images for: Heterochromatin reorganization associated with the transcriptional reprogramming under viral infection in Arabidopsis
Source: Nucleic Acids Res. 2026 Apr 17;54(7):gkag348. doi: 10.1093/nar/gkag348 (PMC13095653; doi:10.1093/nar/gkag348)

# Supplementary Figure 10

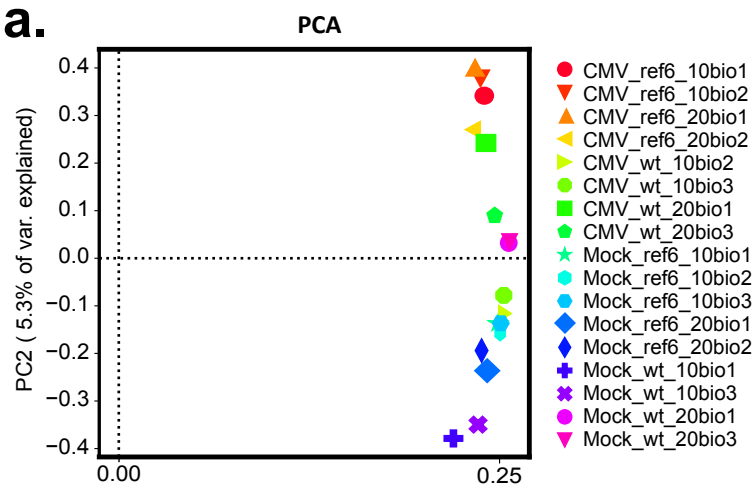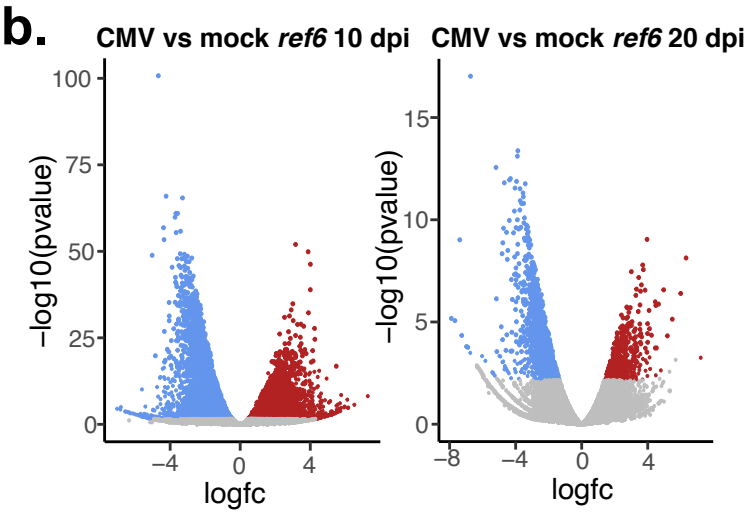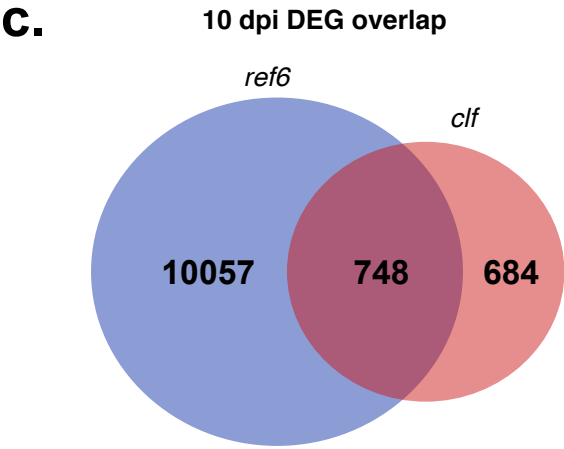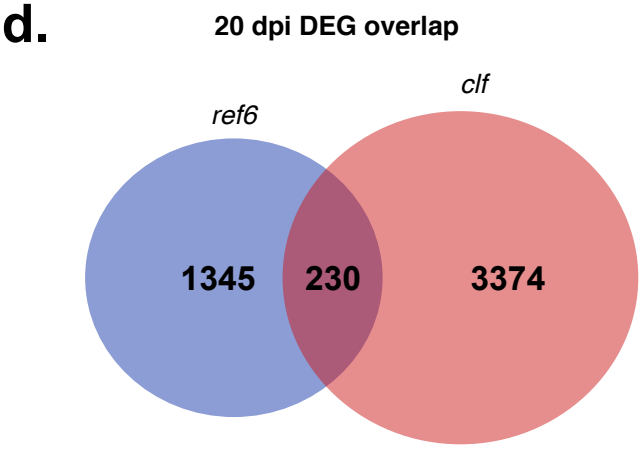

Supplement: gkag348_Supplemental_Files [file gkag348_supplemental_files.zip › Supp_Figure_10.pdf]

# Supplementary Figure 11

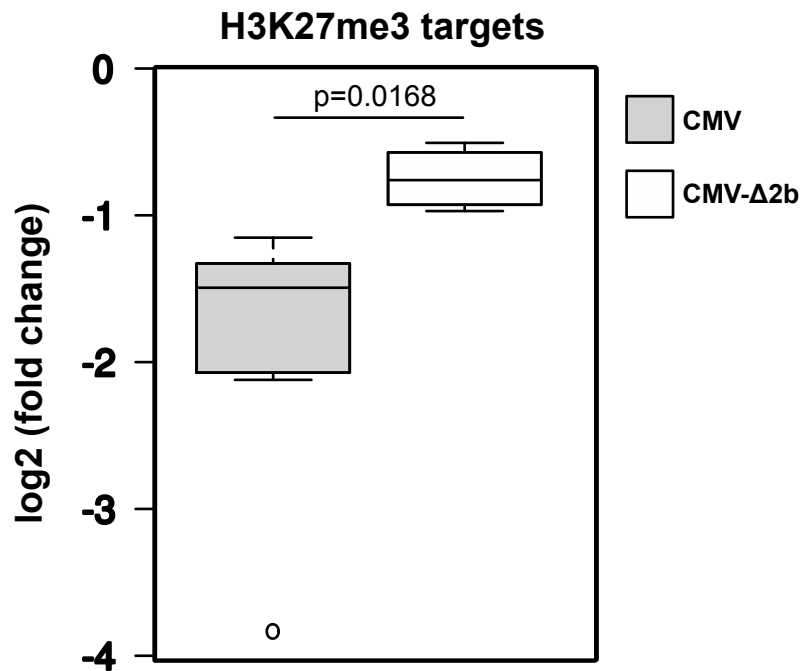

Supplement: gkag348_Supplemental_Files [file gkag348_supplemental_files.zip › Supp_Figure_11.pdf]

# Supplementary Figure 1

a.

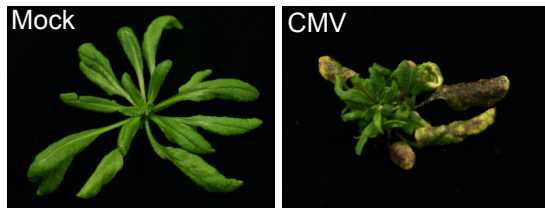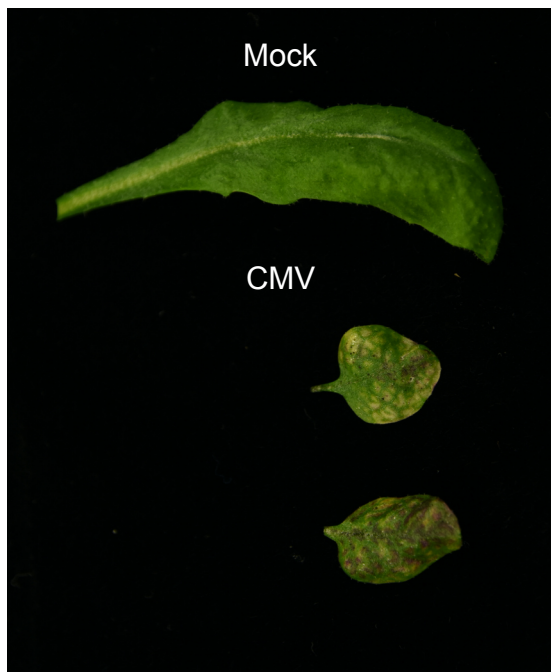

b.

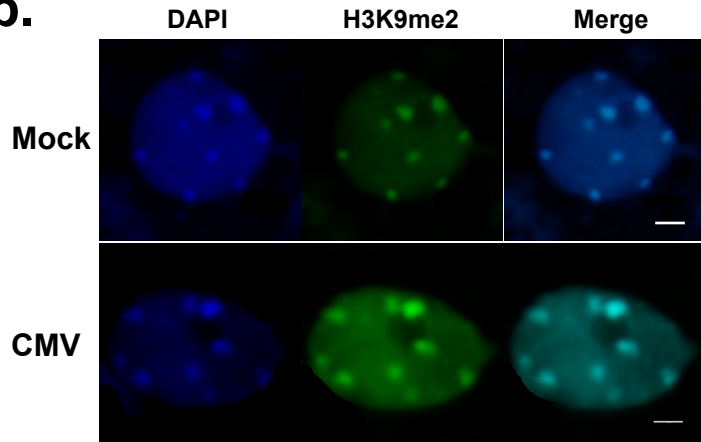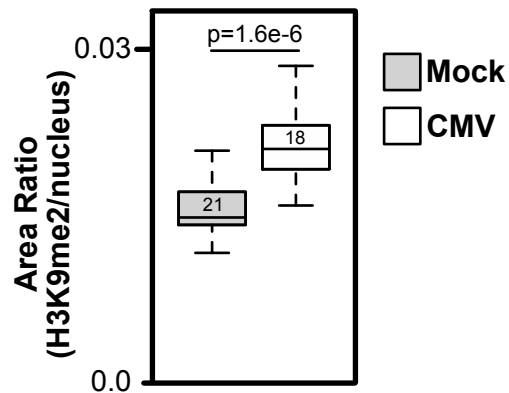

Supplement: gkag348_Supplemental_Files [file gkag348_supplemental_files.zip › Supp_Figure_1_v2_revision_NAR.pdf]

# Supplementary Figure 2

a.

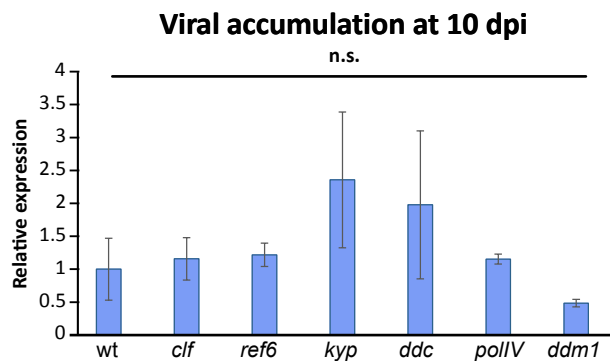

b.

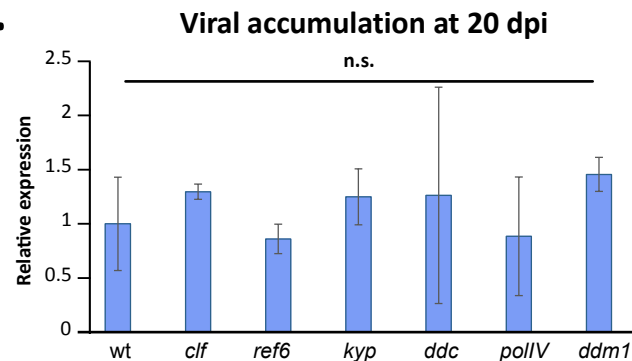

c.

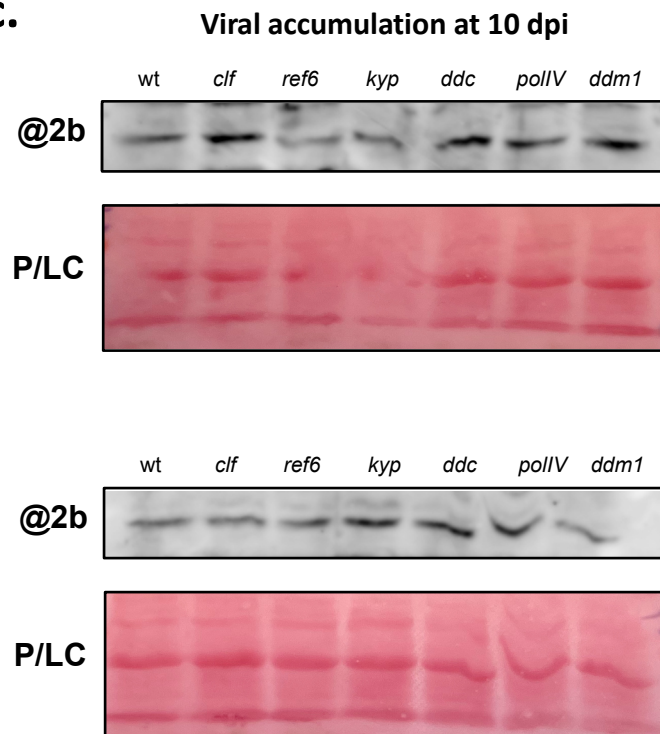

d.

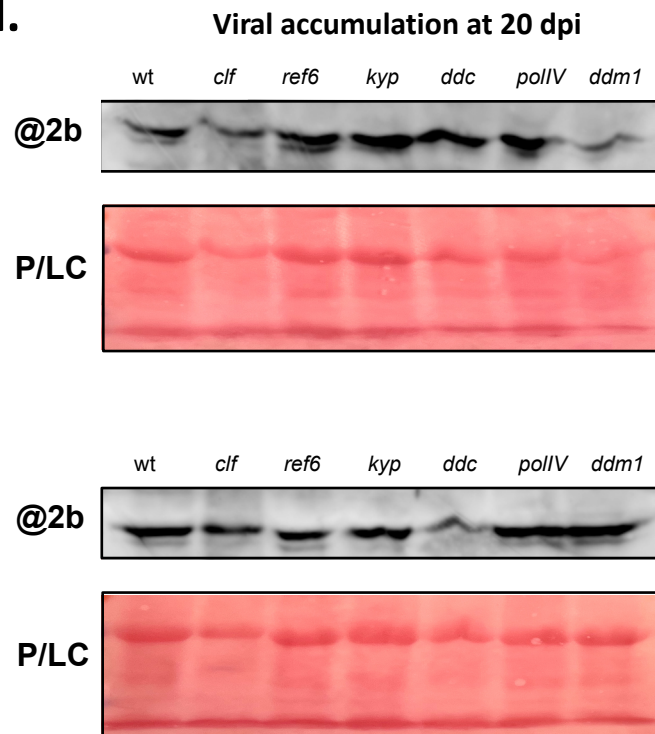

Supplement: gkag348_Supplemental_Files [file gkag348_supplemental_files.zip › Supp_Figure_2_V2_revision_NAR.pdf]

# Supplementary Figure 3

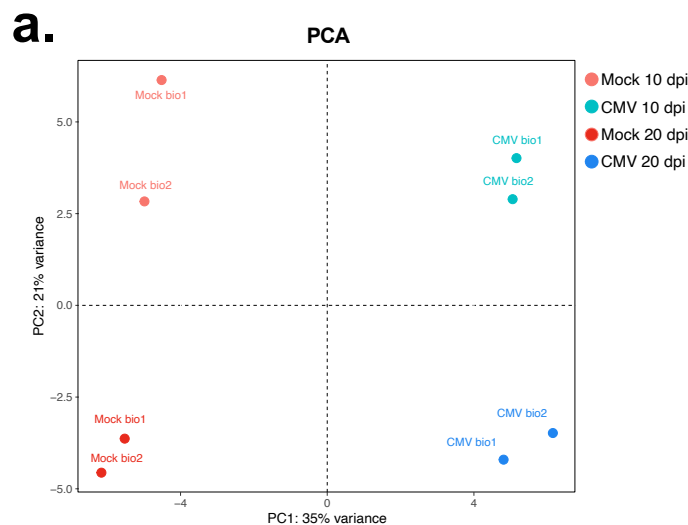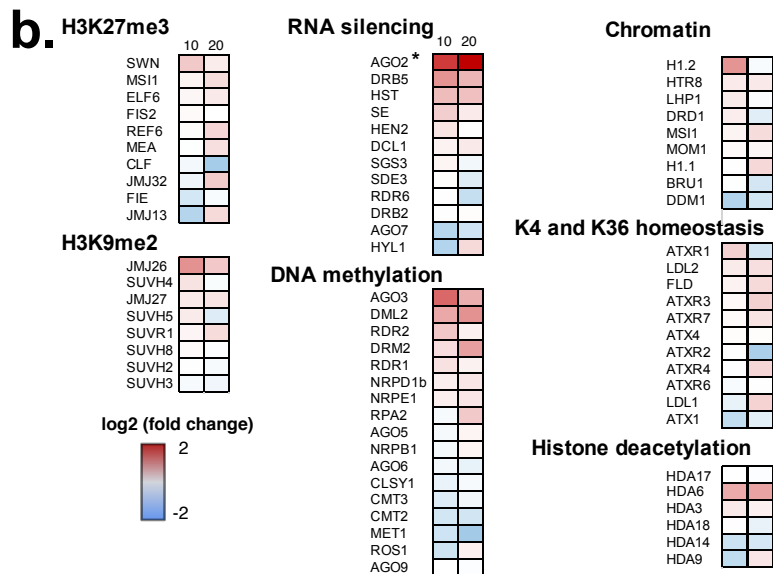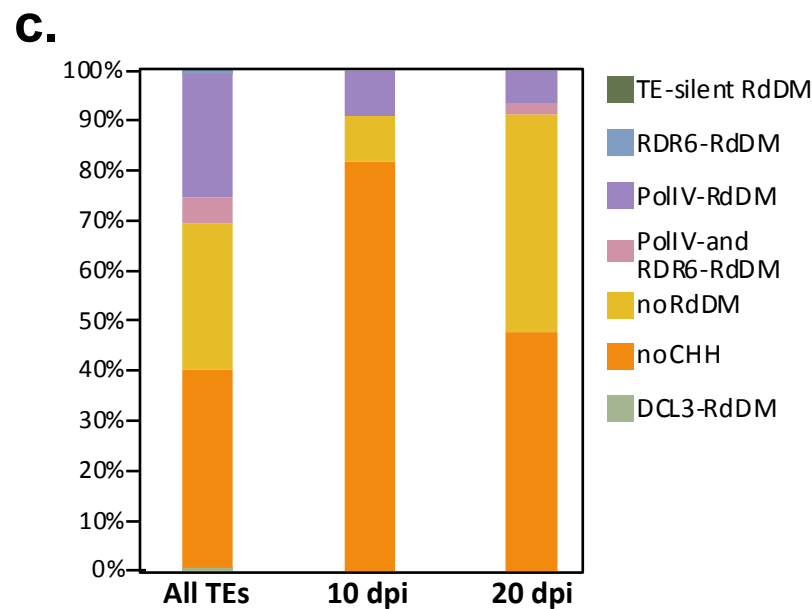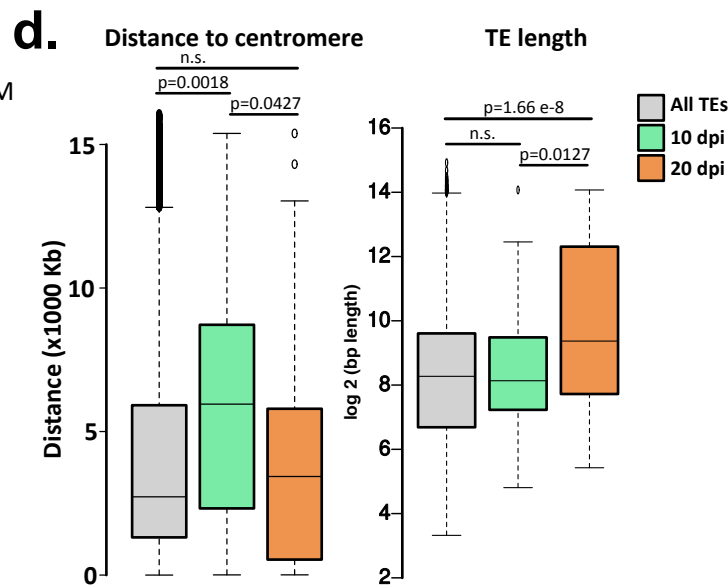

Supplement: gkag348_Supplemental_Files [file gkag348_supplemental_files.zip › Supp_Figure_3_v2_revision_NAR.pdf]

# Supplementary Figure 4

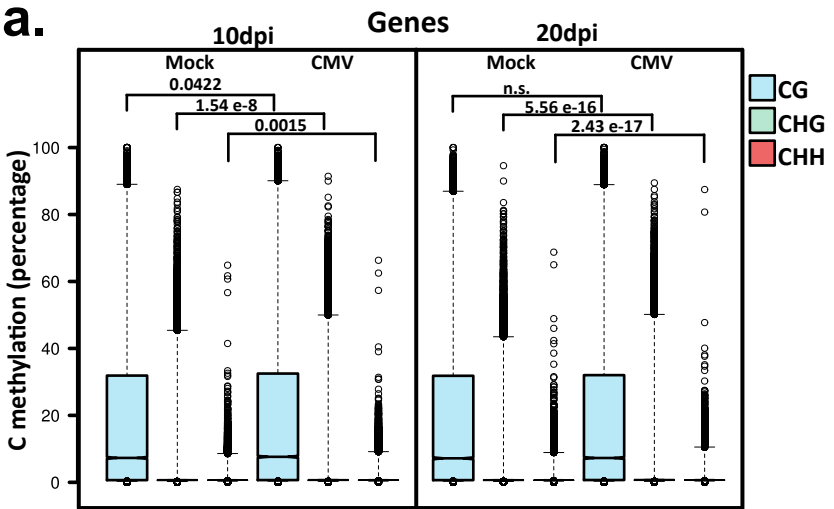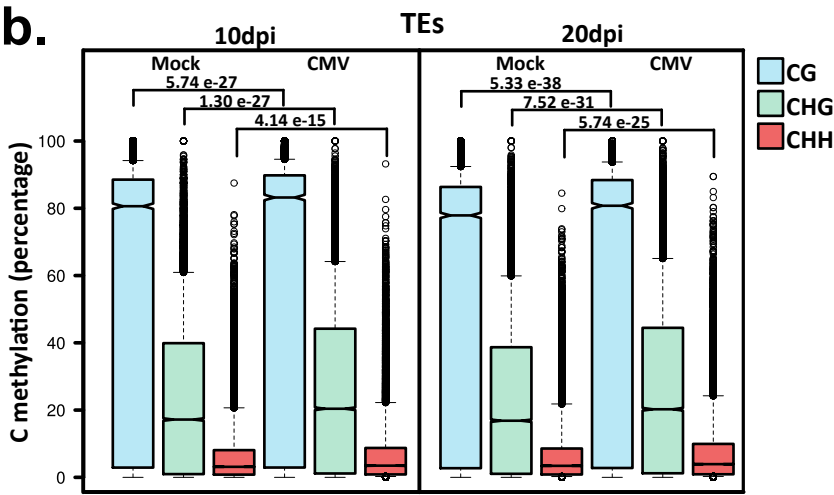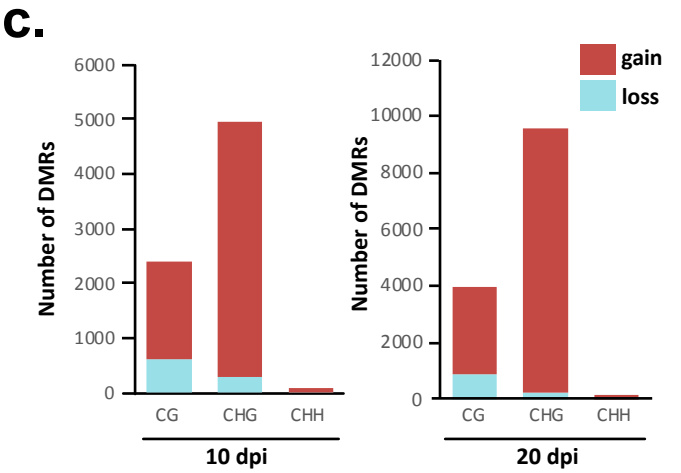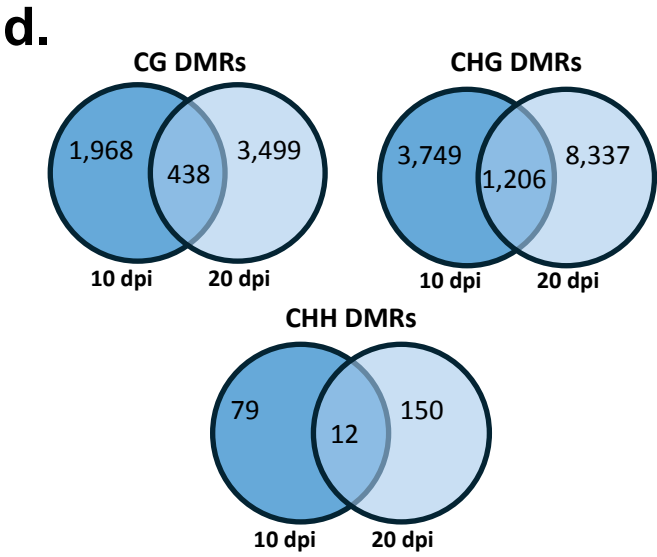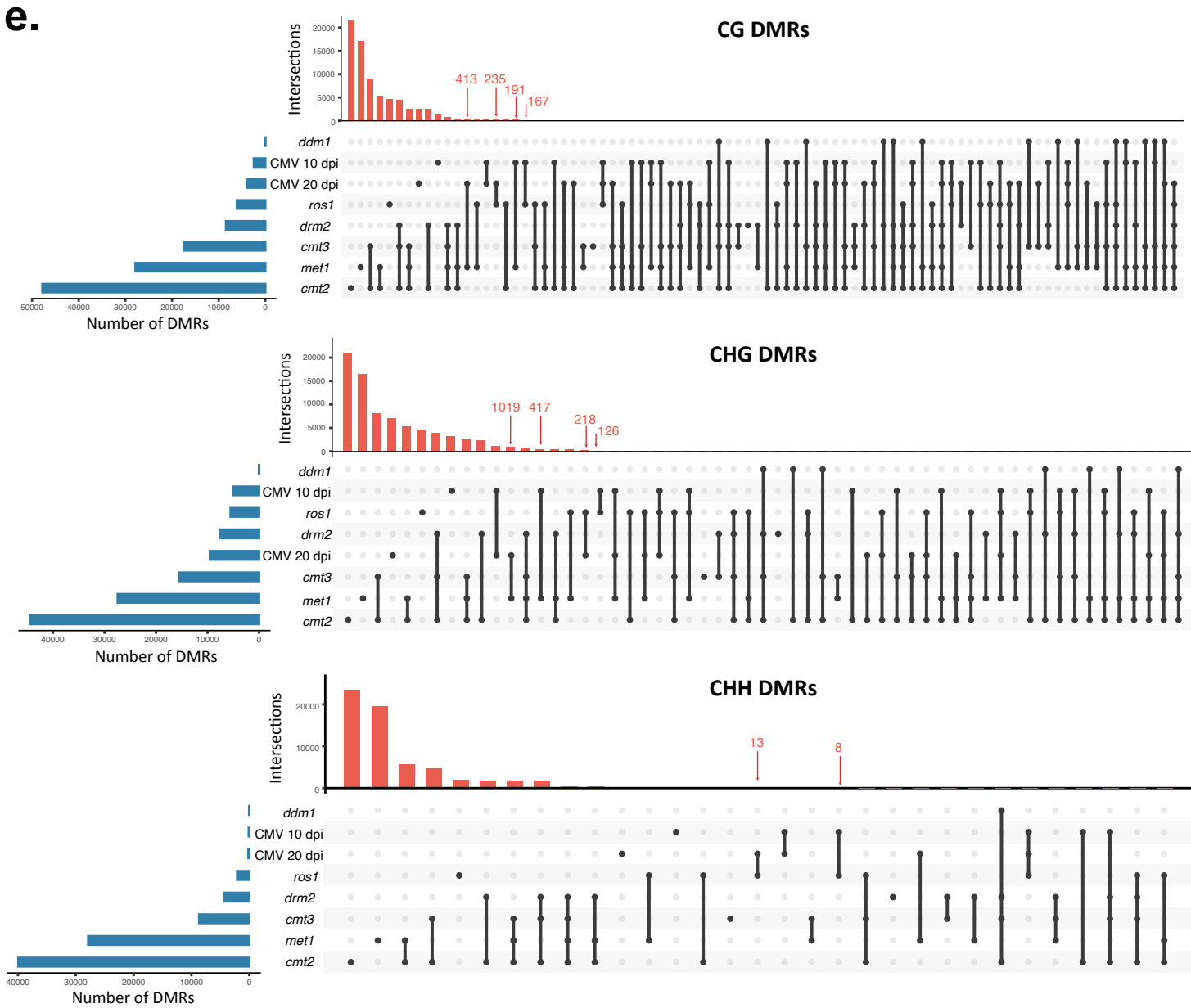

Supplement: gkag348_Supplemental_Files [file gkag348_supplemental_files.zip › Supp_Figure_4_v2_revision_NAR.pdf]

# Supplementary Figure 5

a.

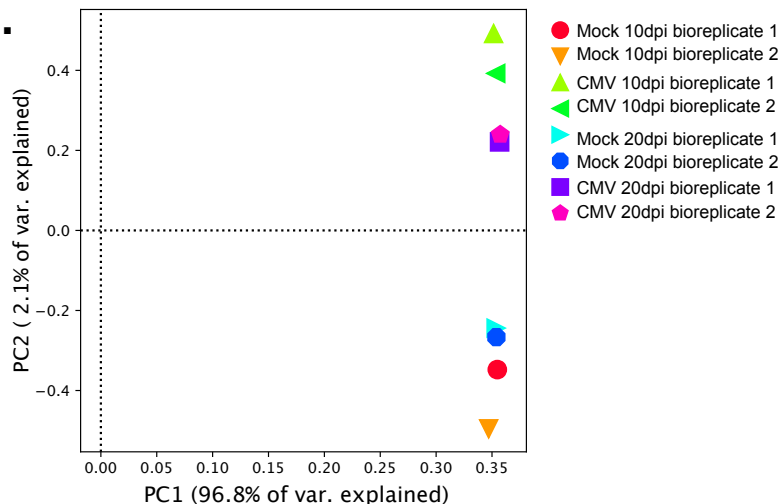

b.

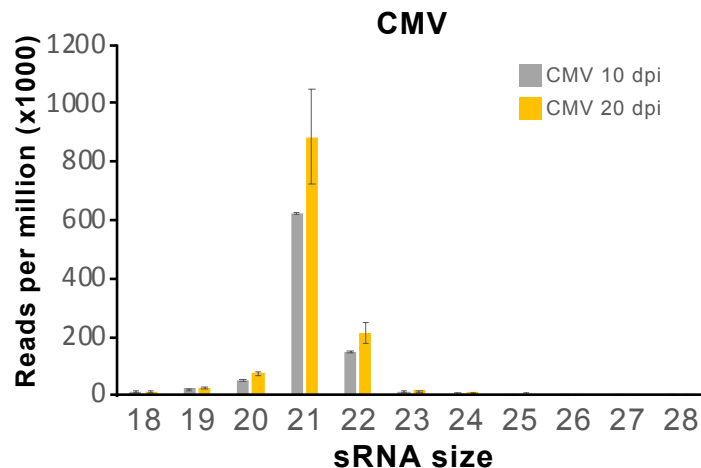

c.

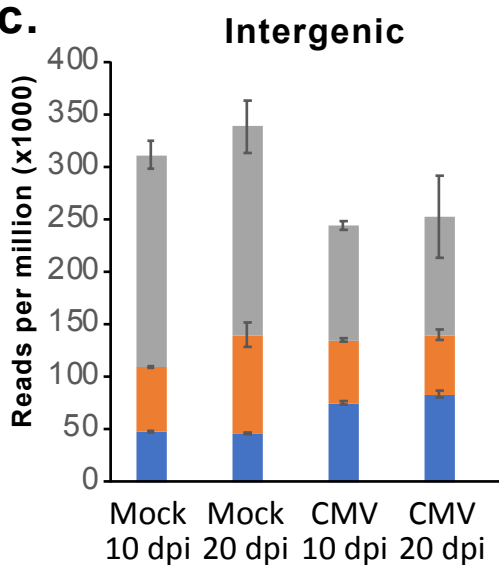

d.

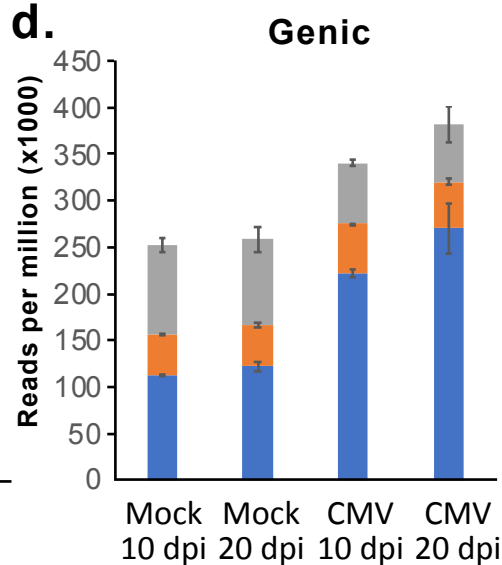

e.

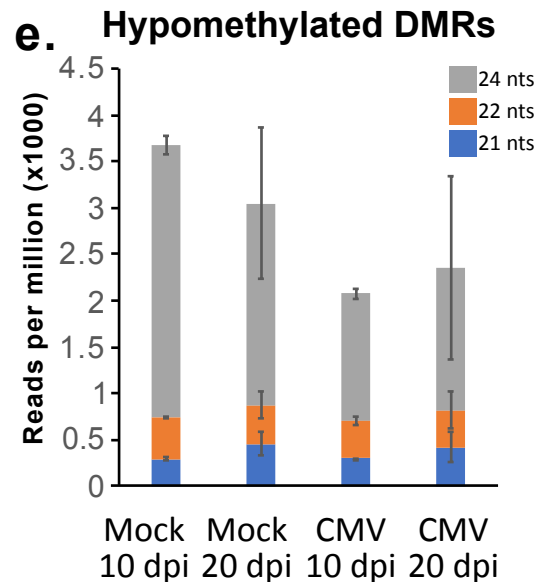

Supplement: gkag348_Supplemental_Files [file gkag348_supplemental_files.zip › Supp_Figure_5.pdf]

# Supplementary Figure 6

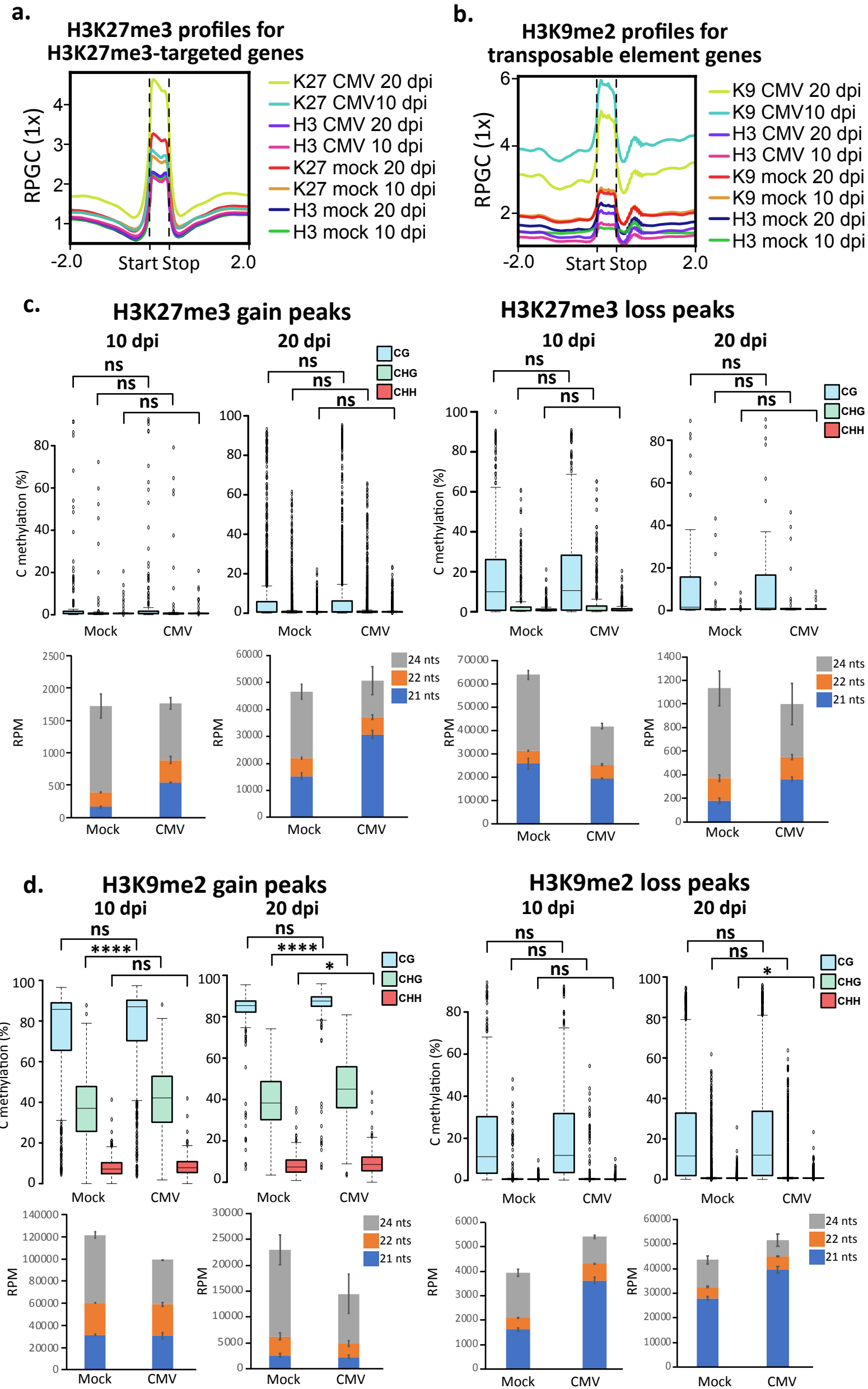

Supplement: gkag348_Supplemental_Files [file gkag348_supplemental_files.zip › Supp_Figure_6_v2_revision_NAR.pdf]

# Supplementary Figure 7

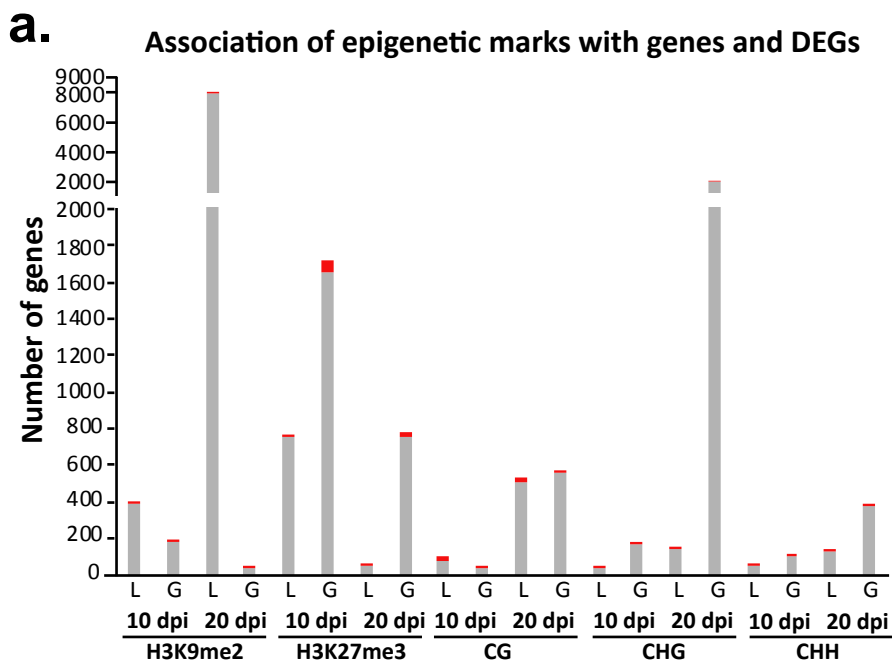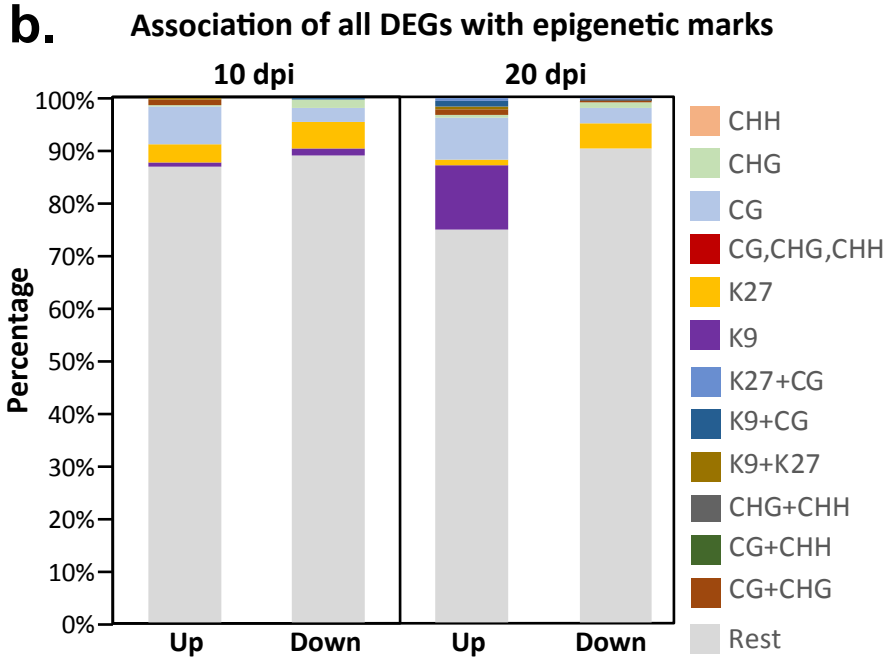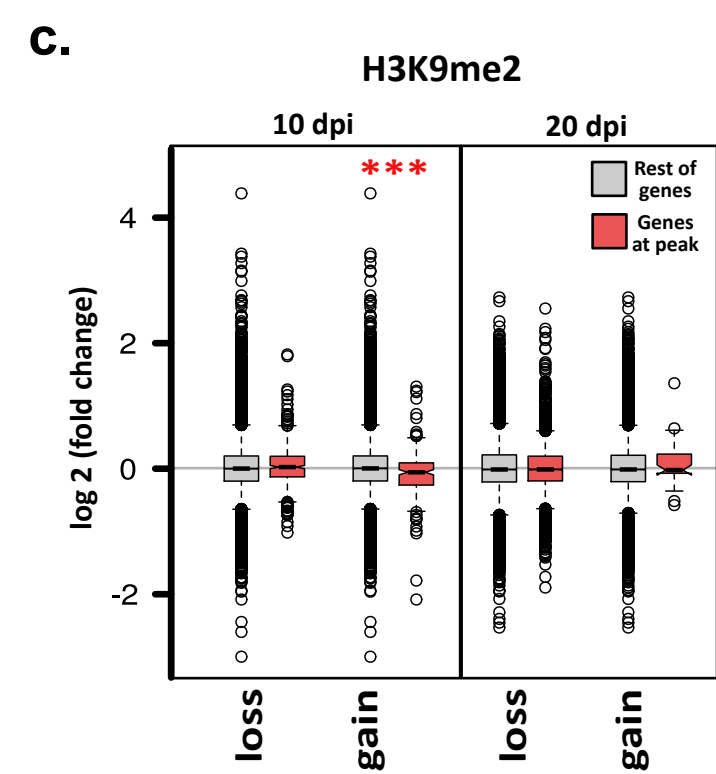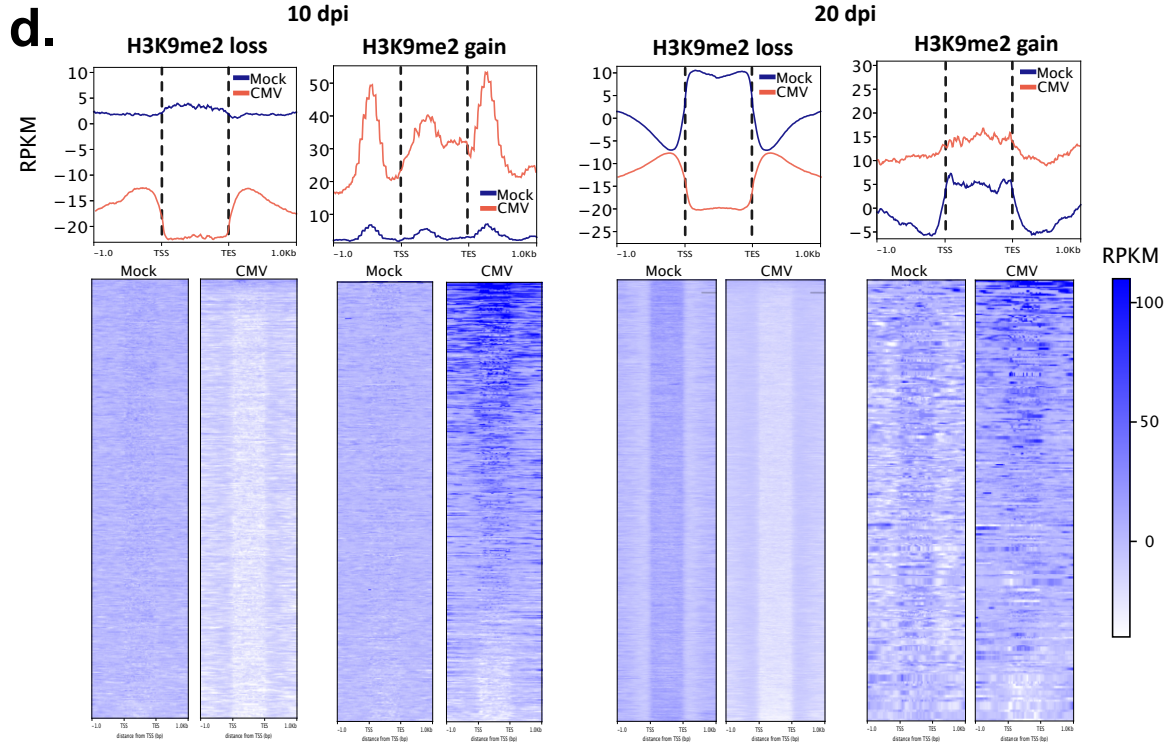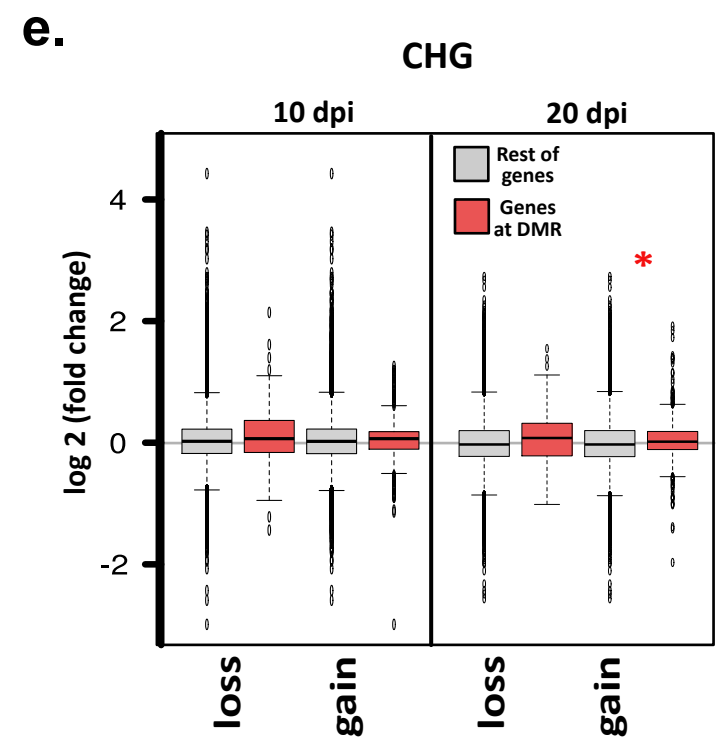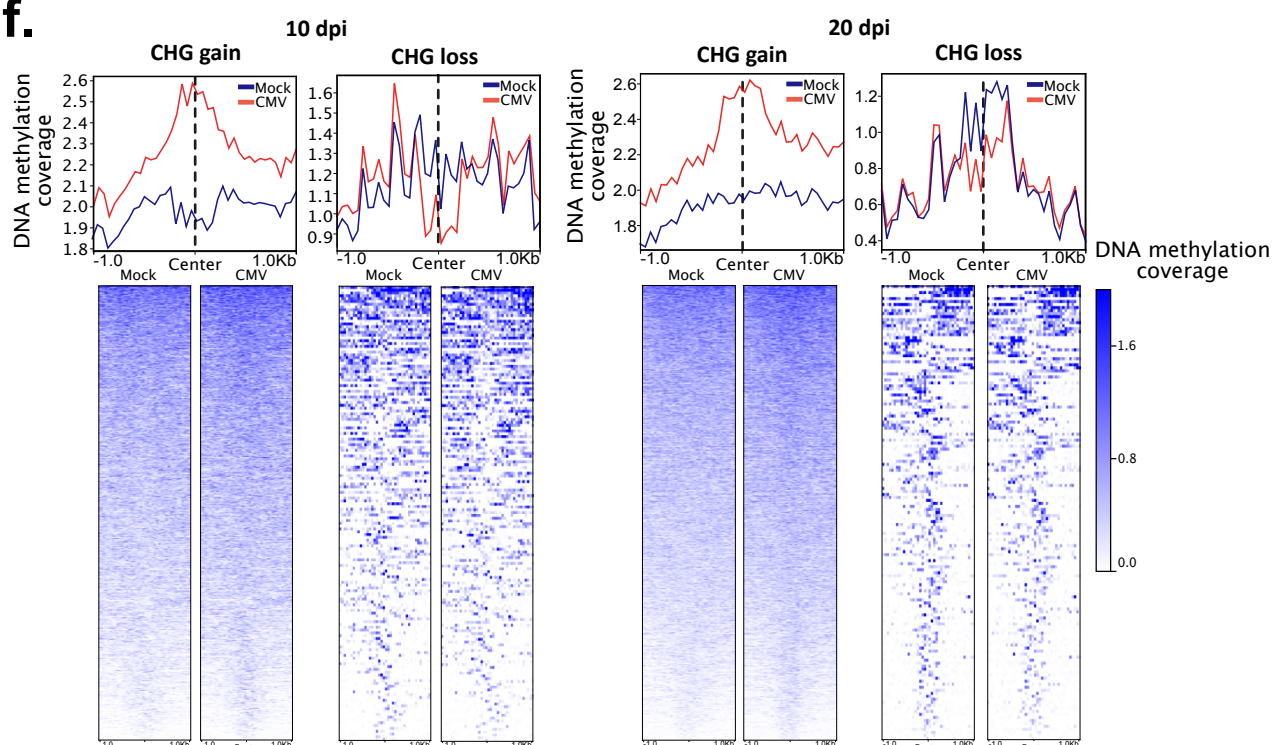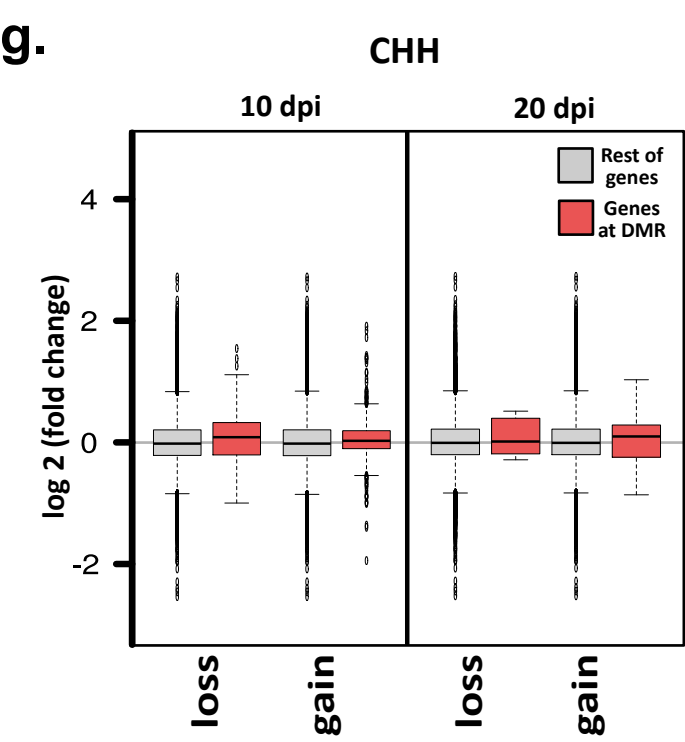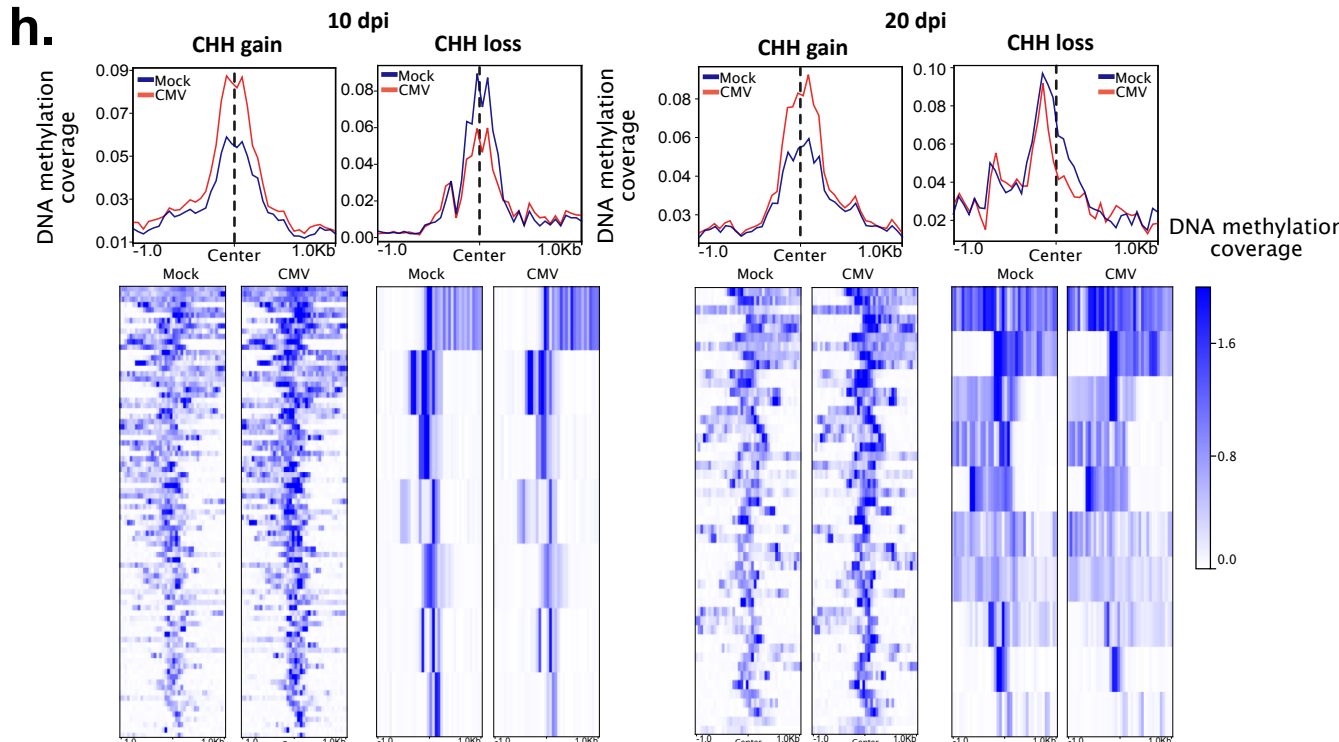

Supplement: gkag348_Supplemental_Files [file gkag348_supplemental_files.zip › Supp_Figure_7_v3_revision_NAR.pdf]

# Supplementary Figure 8

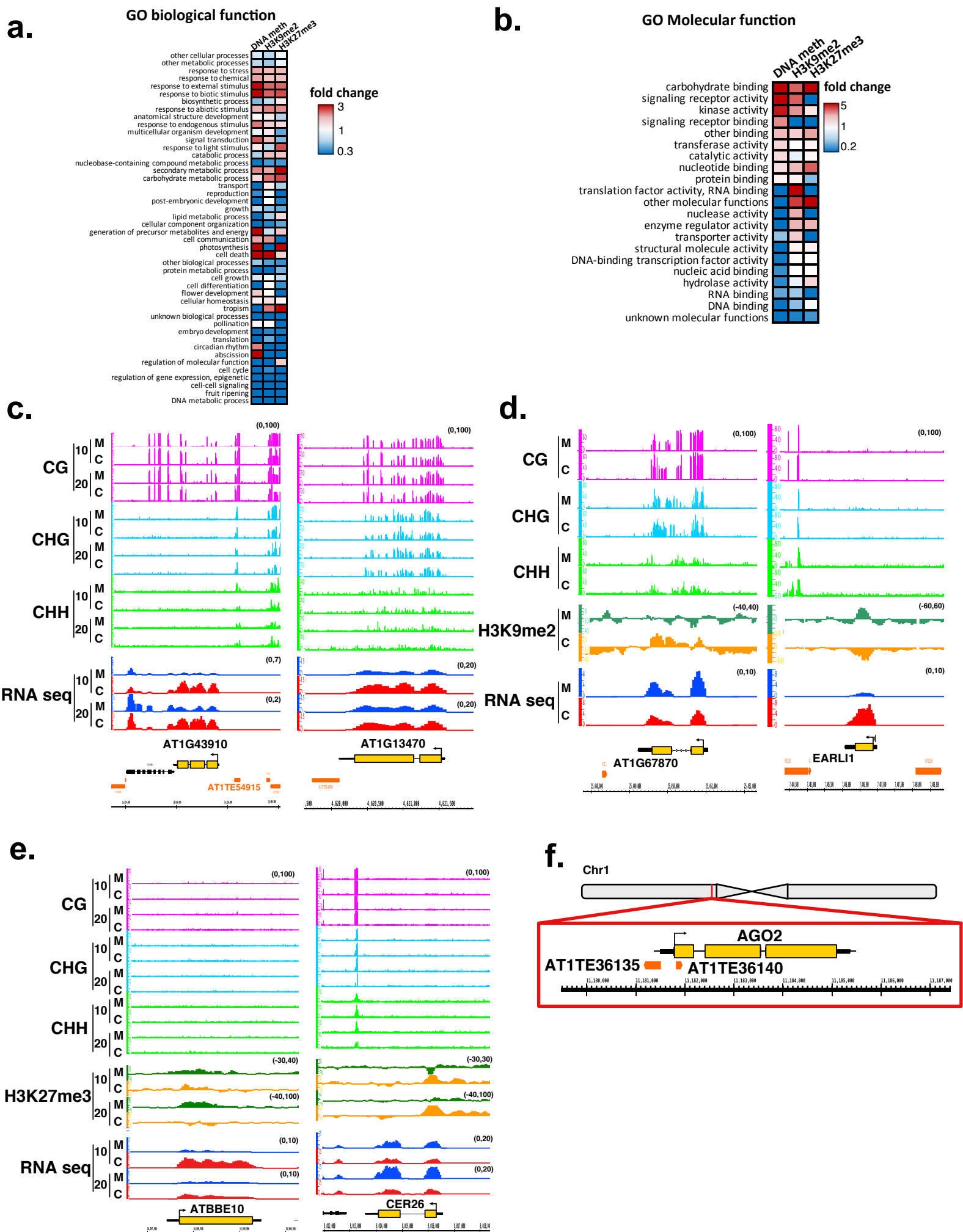

Supplement: gkag348_Supplemental_Files [file gkag348_supplemental_files.zip › Supp_Figure_8.pdf]

# Supplementary Figure 9

**a.**

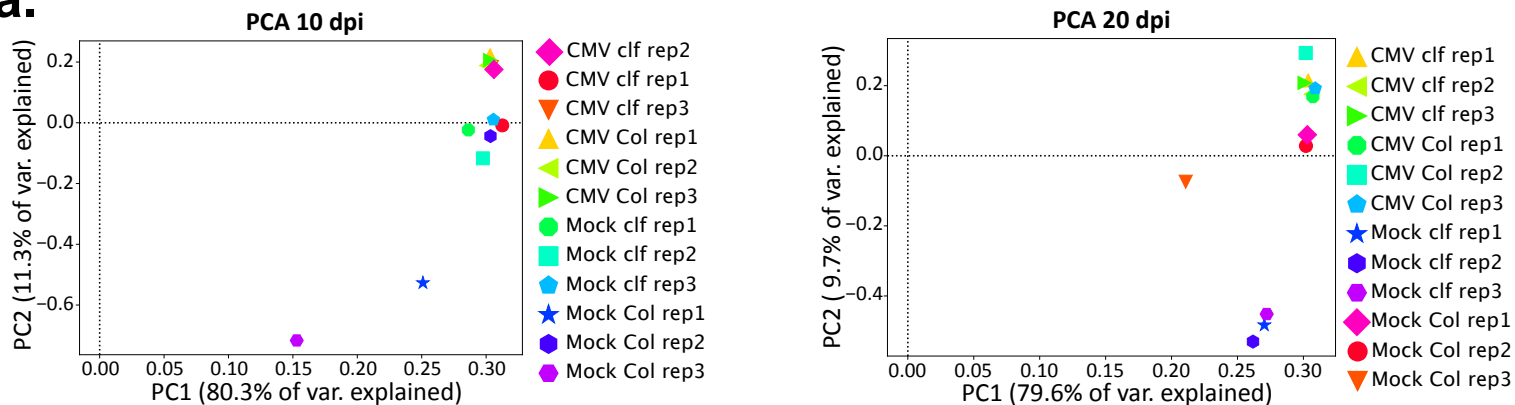

**b.**

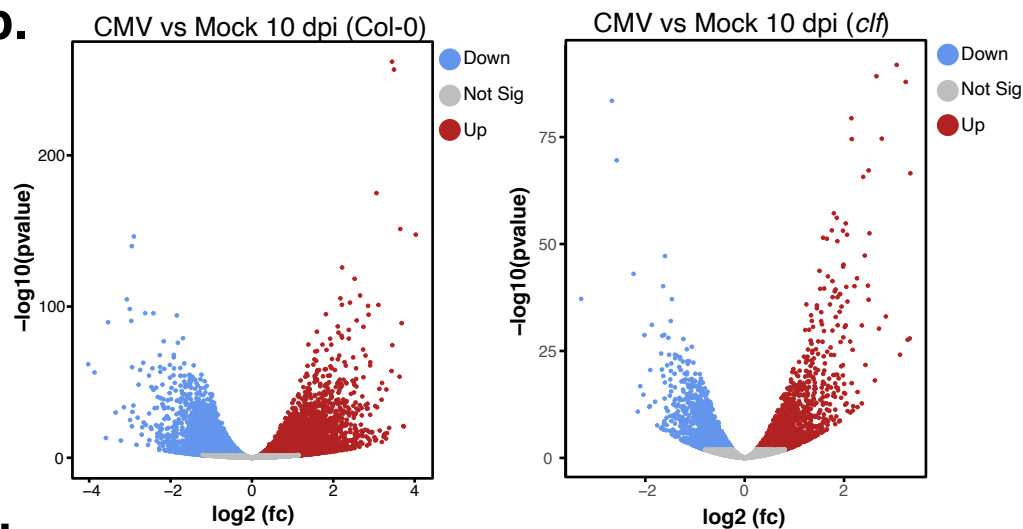

**c.**

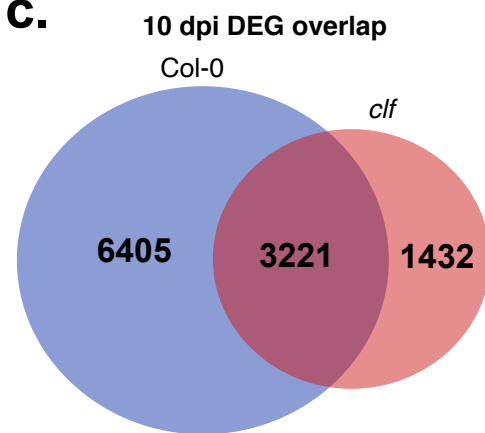

**d.**

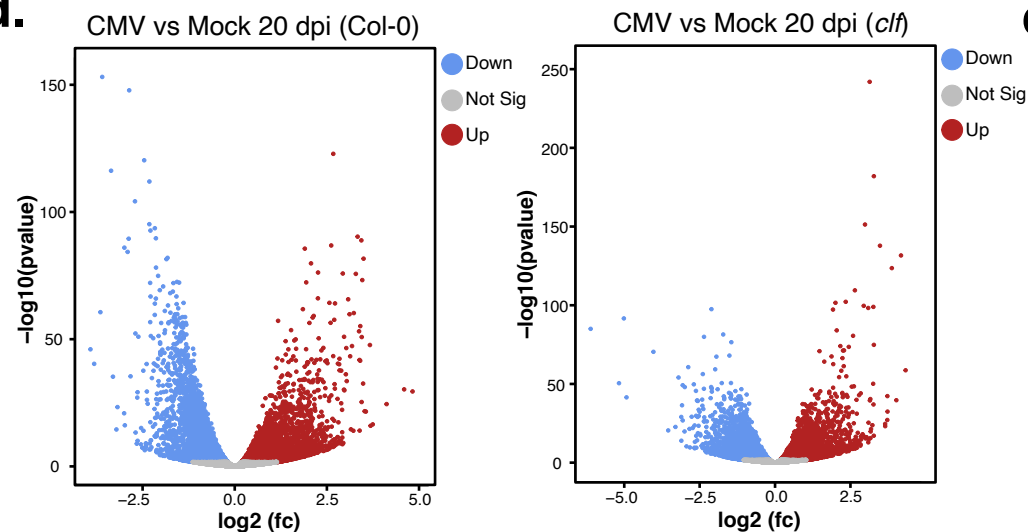

**e.**

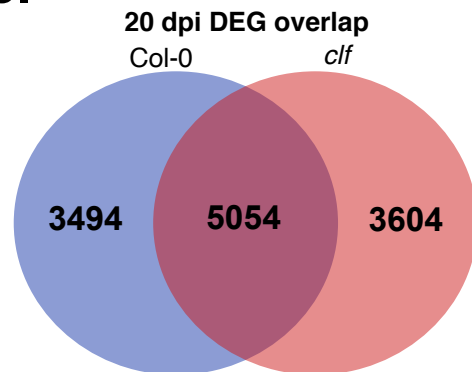

Supplement: gkag348_Supplemental_Files [file gkag348_supplemental_files.zip › Supp_Figure_9.pdf]
